# Bio-oil Fractionation According to Polarity and Molecular Size: Characterization and Application as Antioxidants

**DOI:** 10.1021/acs.energyfuels.4c02641

**Published:** 2024-09-25

**Authors:** Isabel Fonts, Cristina Lázaro, Alfonso Cornejo, José Luis Sánchez, Zainab Afailal, Noemí Gil-Lalaguna, Jesús María Arauzo

**Affiliations:** † Aragon Institute for Engineering Research (I3A), Thermochemical Processes Group (GPT), University of Zaragoza, 50018 Zaragoza, Spain; ‡ Institute for Advanced Materials and Mathematics (INAMAT^2^) - Department of Sciences, Public University of Navarra, 31006 Pamplona, Spain

## Abstract

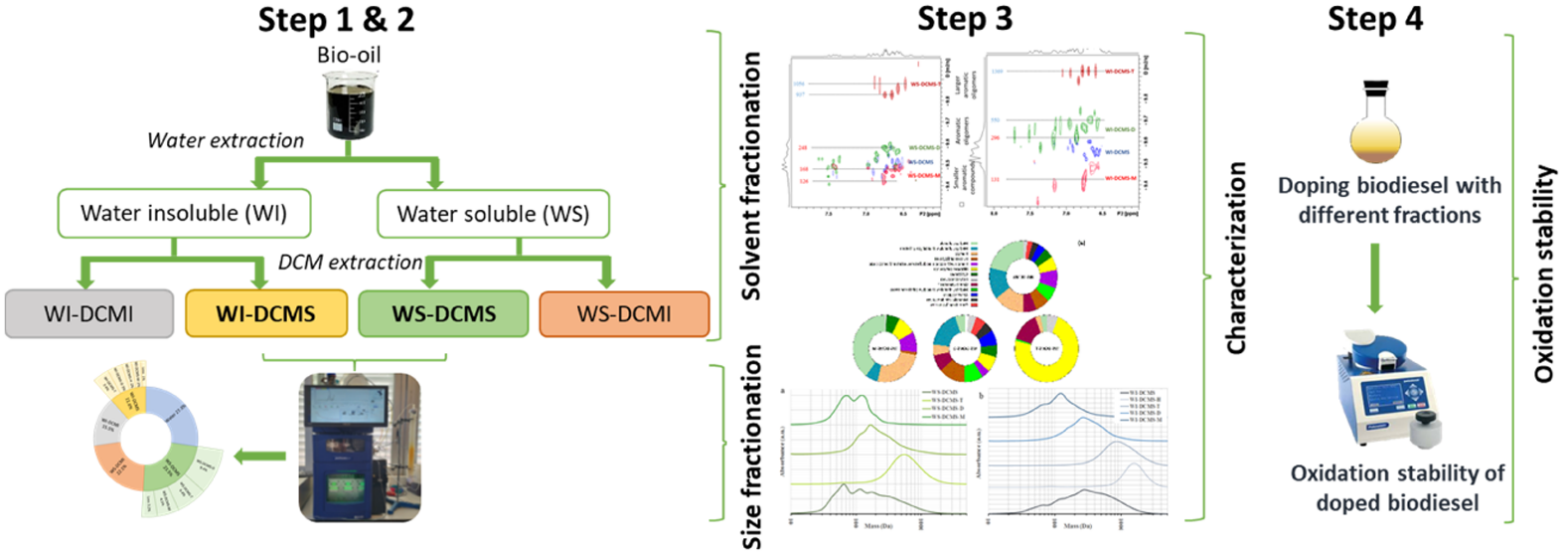

Bio-oil obtained from biomass pyrolysis has great potential for several applications after being upgraded and refined. This study established a method for separating bio-oil into different fractions based on polarity and molecular size to extract phenolic and polyphenolic compounds with antioxidant properties. The fractions were analyzed using various spectroscopic and chromatographic techniques, such as GC/MS, FTIR, UV–vis, SEC, DOSY-NMR, ^13^C-NMR, and ^31^P-NMR. The antioxidant properties of these fractions were tested by examining their ability to improve the oxidative stability of biodiesel. The results strongly connected the bio-oil’s chemical functionalities and antioxidant power. During solvent fractionation, dichloromethane could extract phenolic structures, which were subsequently size-fractionated. The subfractions with lower molecular weight (in the order of monomers and dimers) outperformed the antioxidant potential of the crude bio-oil. Heavier subfractions from dichloromethane extraction did not show good antioxidant abilities, which was related to the low hydroxy group content. After solvent extraction, phenolic oligomers remained in the water-insoluble/dichloromethane-insoluble fraction, which showed good antioxidant potential despite its low solubility in biodiesel.

## 1 Introduction

Approximately 75 wt % of the pyrolysis bio-oil derived from lignocellulosic biomass consists of organic compounds resulting from devolatilization, cracking and thermal ejection of the main biomass components (cellulose, hemicellulose, lignin, and extractives), as well as secondary reactions of the primary pyrolysis products. These compounds encompass a diverse array of chemical families, including aldehydes, ketones, acids, furans, phenols, methoxy phenols, sugars and oligomers.^1^ The broad spectrum of functional groups in the bio-oil compounds positions bio-oil as a promising feedstock for the chemical industry, serving the production of chemical products or fuels.^2^ Consequently, the production of drop-in fuels through bio-oil hydrodeoxygenation has garnered significant attention in recent decades. An alternative approach for fuel production involves coprocessing bio-oil (whole, fractionated, or partly upgraded) with petroleum fractions in conventional oil.^3,4^ Beyond fuels, the scientific community has also focused on producing chemicals from refined bio-oil through separation, reaction, or both over the past few decades.^2^

Regarding the production of chemicals, the European Commission published in 2020 the Chemical Strategy for Sustainability: Toward a Toxic-Free Environment,^5^ which aims to protect citizens and the environment better and boost innovation for safe and sustainable chemicals. Cleaner industrial processes and technologies are required for a green transition. In this sense, bio-oil is a potential resource for obtaining many added-value products, such as fertilizers, pesticides, wood preservatives, resins, antioxidants, carbon fiber, alkylphenols, food additives, asphalt emulsions, as well as base chemicals such as acetic acid, hydroxyacetone, hydroxyacetaldehyde, methanol or levoglucosan.^2^ The market analysis performed by Pinheiro Pires et al.^2^ concluded that the production of polymers from bio-oil should be the highest priority application due to their high added value in the market, while the second priority use should be the development of small oxygenated molecules to be utilized as solvents, chemicals and fuel additives. However, up to date, the routes dedicated to the production of these chemicals have been much less studied than biofuel production from lignocellulosic pyrolysis oils. According to the Scopus database, the production of biofuels from bio-oil is, by far, the most studied application, with more than 3800 research publications focused on the production of biofuels in the last three decades, being around 800 those aimed at the production of drop-in fuels via hydrodeoxygenation of bio-oil and around 300 dealing with coprocessing of bio-oil in conventional oil refineries. On the other hand, the number of published works on producing valuable chemicals from bio-oil is below 150, evidencing the need to expand the number of scientific works on this topic.

Aiming at recovering or producing chemicals from bio-oil, phenolic compounds coming from lignin are an interesting fraction since they make up an important portion of bio-oil (up to 32 wt %)^2^ and provide unique opportunities to obtain specific aromatic and cycloalkane hydrocarbons which are not available via other sustainable processes.^6,7^ Phenolics in bio-oil have also been successfully tested to produce phenolic resins and adhesives^8,9^ or to produce polyols from which polyurethane foams have been later produced.^10^ In biological fields, phenolic compounds typically present in bio-oil have been demonstrated to act as antioxidants in cellular processes^11^ and lipid peroxidation.^12^

In the field of biofuels, commercial antioxidants primarily consist of petroleum-derived synthetic compounds. Biodiesel is doped (at concentrations of 200–1000 ppm) with synthetic phenolic compounds like *tert*-butylhydroquinone (THBQ), pyrogallol (PG), butyl-hydroxytoluene (BHT) or propyl gallate (PG) to improve its oxidation resistance. However, replacing these synthetic additives with naturally occurring substances aligns better with fuel sustainability goals. Bio-oil fractions have also demonstrated antioxidant activity in this field, improving biodiesel’s resistance to oxidation.^13,14^ The hydroxy group in phenols may act as free-radical scavengers (hydrogen donor) in the radical-mediated oxidation of unsaturated fatty acid methyl ester (FAME)^15,16^ or as oxygen scavengers preventing oil oxidation,^15,17^ similar to other commercial antioxidants used in applications like food packaging, drugs and cosmetics.

Therefore, new and sustainable production pathways may be developed from biomass within this industrial sector. Nonetheless, similar to any industry pursuing specific platform chemicals from biomass, producing antioxidants from bio-oil will necessitate the isolation of compounds. This is because bio-oil encompasses a broad and heterogeneous array of chemical entities, including undesirable compounds that may exert adverse effects. In fact, bio-oil is inherently unstable during storage owing to its high oxygen content.

Not only chemical functionalities but also molecule size and complexity should be considered when defining the application of phenolic fractions.^18^ According to their molecular size, phenolics in bio-oil can be grouped into light phenolic compounds originated by cracking and evaporation (boiling temperature lower than the pyrolysis temperature)^1^ and heavier compounds commonly known as lignin oligomers, which are thermally ejected from lignin during pyrolysis.^19^ The distribution of molecular masses of these phenolic compounds may vary between 94 Da for phenol and more than 1500 Da for pyrolytic oligomers.^19^

Separating phenolic compounds according to their molecular size could be an interesting strategy to solve some drawbacks caused by the heavier oligomers during, for example, hydrodeoxygenation treatment of bio-oil.^20^ Regarding its use as antioxidants, previous work in our group highlighted that the concentration of monomeric phenols cannot fully explain the antioxidant potential of bio-oil fractions. Still, the heaviest fraction could also play a significant role.^21^ Some other studies in the literature point out that most of the simplest (monomeric) phenols usually remain inactive as antioxidants for fatty acid esters due to the predominance of hydrogen bonding (kinetic bonding effect). Effective antioxidants for fatty acid esters may include bifunctional molecules and likely dimers having separate hydrogen-bonding and radical-quenching sites, such as those containing catechol and guaiacol groups. Further research is required to definitively identify the most active oxidation inhibitors, allowing the development of novel additives to help replace synthetic materials currently used.^22^

Different strategies based on solvent fractionation schemes with organic and inorganic solvents have been successfully applied to isolate various fractions of pyrolysis bio-oil.^23^ The solvent fractionation scheme developed by Oasmaa et al.^24^ allows the separation of the phenolic compounds from pyrolysis bio-oil into low and high molecular weights. In the same way, Wang et al.^25^ developed a multistep procedure based on solvent extractions with organic and inorganic solvents, which allows the separation of phenolics according to their molecular weight into four fractions after successive extraction, filtration and removal of the solvents. Despite the laborious separation procedure applied,^25^ the fractions obtained presented a wide distribution of molecular weights, including, in two of the four separated fractions, phenols from monomers to >10-mers. To solve this issue, preparative size-exclusion chromatography (preparative-SEC) is another reliable and systematic separation methodology that allows the separation of molecules according to differences in size and structures, providing fractions with narrower molecular weight distributions. This technique has already been successfully applied to separate coal liquids, petroleum residues, soots, biomass tars and humic substances.^26^

Exhaustive characterization of the fractions obtained after bio-oil fractionation is essential for further application. For example, phenolic fractions can behave differently in terms of conversion and selectivity to products during HDO treatment depending on the abundance of their hydroxy groups and the type of other substituents (alkyl or methoxy), as well as depending on their molecular weight.^27,28^ Besides chromatographic techniques, Nuclear Magnetic Resonance (NMR) has become a powerful tool in characterizing bio-oils. ^31^P-NMR has been widely used in the determination of the number of hydroxy groups after derivatization with a phosphorus reagent,^29^ allowing not only its quantification but also its assignation to the different aromatic types of compounds that are present in bio-oil.^30^ Quantitative ^13^C-NMR has also been widely used to characterize pyrolysis oils to gain insight into the quantitative distribution of the different functional groups.^31,32^ Diffusion Ordered Spectroscopy (DOSY-NMR) correlates the chemical shifts with the diffusion coefficient (D) and consequently with the molecular weight,^33^ so it can be seen as a pseudotechnique that combines the NMR and the SEC techniques.^34^ DOSY has shown good efficiency in the characterization of standard mixtures of phenolic compounds.^35^ Still, it has not found much application in estimating the apparent mass of complex samples, except in a few studies dealing with bio-oil or fractions arising from lignin depolymerization.^36−38^

Synchronous UV-fluorescence spectroscopy is another technique used to characterize different substances, such as drugs^39^ or polycyclic aromatic hydrocarbons,^40^ as well as to authenticate SARS-CoV-2 vaccines from different manufacturers.^41^ In biomass thermochemical processing, some researchers have recently demonstrated the utility of this technique to evaluate the effect of the reaction time of a catalytic treatment on the degree of polymerization of the bio-oil phenolic fraction.^42^

The main contribution of this study to the state of the art of bio-oil exploitation lies in the detailed characterization of bio-oil fractions and the demonstrated efficacy of some isolated fractions as antioxidant additives for biodiesel. By employing a combination of solvent extraction and preparative size-exclusion chromatography (SEC), specific phenolic fractions that significantly enhanced the oxidative stability of biodiesel were isolated. This approach provides a deeper understanding of the antioxidant properties of bio-oil components and opens new pathways for extracting sustainable chemicals from bio-oil.

## 2 Materials and Methods

### 2.1 Raw Bio-oil

The bio-oil used in this study was produced by the company BTG (The Netherlands) from pine wood using their proprietary Rotating Cone Reactor technology (BTG Bioliquids). A pyrolysis temperature of 510 °C was used in the reactor, while the pyrolysis vapors were condensed in one step at 40 °C. When bio-oil was received from the company, it was stored at −24 °C before the performance of this work. The elemental analysis (CHN628 Series from LECO) of the raw bio-oil showed that, on a moisture basis, it contained 43.4 wt % of C, 7.4 wt % of H, 0.15 wt % of N and 49.1 wt % of O (calculated by difference). The water content of the raw bio-oil determined by Karl Fischer titration was 27.3 wt %.

### 2.2 Fractionation of Bio-oil

Bio-oil was fractionated following two subsequent procedures: (i) first solvent extraction and then (ii) preparative-SEC of some of the fractions extracted in the previous stage. The whole fractionation scheme is explained in depth in the Supporting Information (Section S1). A summary of the procedure is included next.

Solvent extraction of bio-oil was carried out with water and subsequently with dichloromethane (DCM) following an adapted method from Oasmaa et al.^24^ Four fractions were obtained: water-soluble/DCM-insoluble (WS-DCMI), water-soluble/DCM-soluble (WS-DCMS), water-insoluble/DCM-soluble (WIS-DCMS) and water-insoluble/DCM-insoluble (WS-DCMI). The two fractions soluble in DCM (WS-DCMS and WI-DCMS) were fractionated by preparative-SEC using a Puriflash 5.125 (Interchim, France), equipped with an Omnifit column (25 mm diameter and 50.5 cm long). The stationary phase consisted of a Bio-Bead S-X3 resin (Bio-Rad Laboratories) that was swollen in DCM overnight. The equipment had an ultraviolet (UV) detector (200–400 nm) and an automatic system to collect the different subfractions. Three phenolic model compounds were used to optimize the size-exclusion separation procedure and to set the elution time intervals: a monomer (phenol, 94.1 g/mol), a dimer (2,2′-methylenebis(6-*tert*-butyl-4-methylphenol), 340.5 g/mol) and a tetramer (1,3,5-trimethyl-2,4,6-tris(3,5-di*tert*-butyl-4-hydroxybenzyl)benzene, 775.2 g/mol). Three subfractions were separated from the WS-DCMS fraction (WS-DCMS-M, WS-DCMS-D and WS-DCMS-T) and four more from the WI-DCMS one (WI-DCMS-M, WI-DCMS-D, WI-DCMS-T and WI-DCMS-H). M-, D- and T- denote monomers, dimers, and tetramers for those compounds eluting at the same time windows as the phenolic model compounds used for the procedure optimization. H- means heavy, representing the biggest compounds that elute before the tetramer model compound.

### 2.3 Chemical Characterization of the Bio-oil Fractions

The characterization of the bio-oil fractions has been carried out using the following techniques:

#### 2.3.1 Gas Chromatography Coupled with Mass Spectrometry and Flame Ionization Detection (GC-MS/FID)

This analytical technique allowed the quantification of the most volatile compounds in the different bio-oil fractions. An Agilent GC/MS/FID (7890*A*/5975C) was used for the analyses. For better identification and quantification, samples were previously derivatized by silylation with N,O-Bis(trimethylsilyl)trifluoroacetamide (CAS 25561-30-2; Sigma-Aldrich). Identification of compounds was performed with the MS signal using spectra in the NIST14 library, whereas the FID signal was used for the quantification using relative response factors (RRF) calculated according to the Effective Carbon Number for silylated compounds.^43^ RRF was applied to obtain the percentage of each compound with respect to the GC-elutable sample. A complete description of the experimental procedure (silylation and analysis conditions) is detailed in Section S2 (Supporting Information), and a list of the RRF calculated for each compound is included in Section S2, Table SI-1. After silylation, and also considering their relatively low molecular weights (Section 3.2.4), M- and D-fractions are expected to be virtually GC-elutable, so, in these cases, the obtained percentages can be understood as mass concentrations with respect to the whole fraction.

#### 2.3.2 Attenuated Total Reflectance–Fourier Transform Infrared Spectroscopy (ATR-FTIR)

ATR-FTIR analyses of the fractions were carried out in a Cary 630 (Agilent) in the 4000–400 cm^–1^ range with a 4 cm^–1^ resolution to observe changes in the functional groups.

#### 2.3.3 Synchronous Excitation UV-Fluorescence Spectroscopy (UV-Fluorescence)

This technique gives an idea of the molecular size distribution of compounds with resonance, so it was useful to evaluate the mass of the aromatic compounds present in the bio-oil fractions and, thus, the performance of the size fractionation. Samples were diluted in ethanol (50 mg/L) and measured in a UV-3600 (Shimadzu, Japan) in the 200–500 nm range at 100 nm/min and a wavelength offset of 20 nm. Three wavelength intervals were set up to integrate the area below the curve: 300 nm for phenol and phenols substituted with side chains, between 300 and 340 nm for oligomers and from 340 nm onward for lignin residues. These assignments were based on the peaks of maximum emission obtained for model phenolic compounds (Table SI-3 in the Supporting Information) and the peak wavelengths observed in the spectra of the samples (286, 327, and 350 nm). Results are shown as the percentage of area below the curve corresponding to each wavelength interval (eq 1).
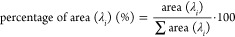
1where area (λ_i_) corresponds to area integrated for λ < 300 nm, for λ = 300–340 nm and for λ > 340 nm.

#### 2.3.4 Size-Exclusion Chromatography (SEC)

Like UV-fluorescence, this technique was used to determine the molecular weight distribution of the bio-oil samples. When coupled with a UV absorbance detector, SEC is particularly useful for measuring aromatics (phenolics), while a refraction index detector (RID) allows more general measurements. The measurements were done on an Agilent 1100 equipped with coupled HR-5 and HR-1 Styragel columns (Waters) as stationary phase at 30 °C and tetrahydrofuran as mobile phase at 1 mL/min. Linear polystyrene standards were used for calibration. Both standards and samples were prepared in THF with a 10 mg/mL concentration. The absorbance of the samples was measured at 254 nm, which is appropriate for detecting phenols.

#### 2.3.5 Nuclear magnetic resonance (NMR)

Nuclear magnetic resonance (NMR) spectroscopy is a versatile technique used in this work with different aims. Quantitative ^13^C-NMR afforded an estimation of the various functional groups present in the isolated fractions, and ^31^P-NMR provided an analysis of the different types of hydroxy groups after the derivation of the sample. Diffusion Ordered Spectroscopy (DOSY) allowed the estimation of the apparent mass and the identification of functional groups in the bio-oil fractions. These analyses were made at 300 K on a Bruker Ascend III spectrometer equipped with a PH-BBI 5 mm probe, at 400 and 101 MHz for ^1^H and ^13^C, respectively. They were processed using Bruker Topspin 3.6.2 software and Dynamics Center 2.6.1. A complete description of the experimental and data analysis procedure is shown in the Supporting Information (Section S2).

### 2.4 Antioxidant Effect of Bio-oil Fractions on Biodiesel Oxidative Stability

The DCM-soluble fractions and those subfractions obtained after their molecular weight fractionation were tested as biodiesel antioxidant additives following a procedure already described by our research group.^44^ To summarize the protocol, lab-made sunflower biodiesel was doped at 1 wt % with the bio-oil fractions, adding DCM as a cosolvent to help in solubilization. DCM was subsequently removed at 40 °C in a rotary evaporator, and the doped biodiesel was centrifuged to remove the insoluble part of the additive, which was gravimetrically measured, thus allowing the calculation of the actual solubilized dosage of the additive (ASD). The oxidation stability of the neat biodiesel and the doped samples was measured with a PetroOXY device (Petrotest Instruments GmbH) according to the ASTM D7545 test method. Briefly, in this test, 5 mL of biodiesel is introduced into the equipment, a pressure of oxygen of 700 kPa is set, and the temperature is increased to 140 °C. The pressure is continuously recorded until the pressure drops 30% over the maximum pressure attained after the heating period. This measured time is the oxidation stability time and indicates the resistance of the sample to be oxidized: the longer the time is, the higher the oxidative resistance. To compare the antioxidant power of each bio-oil fraction, the parameter AntiOxP was defined considering both the increase in the oxidation stability time concerning neat biodiesel and the solubility of the bio-oil fraction in biodiesel (eq 2).
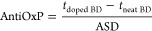
2where *t*_doped BD_ is the oxidation stability time (min) measured for the biodiesel doped with bio-oil fractions, *t*_neat BD_ is the oxidation stability time (min) measured for the neat biodiesel, which serves as blank and ASD (wt %) is the actual solubilized dosage of each bio-oil fraction in biodiesel. As the difference in time is divided by ASD, the AntiOxP parameter is useful for comparing the antioxidant potential of compounds that really act as soluble additives in biodiesel. This means that if two bio-oil fractions, A and B, obtain the same AntiOxP value but the ASD of fraction A is smaller than the ASD of fraction B, then the antioxidant power of fraction A is greater.

For comparison purposes, the oxidation stability of biodiesel doped with a known synthetic phenolic antioxidant additive, BHT (butyl-hydroxytoluene), at dosages <1 wt % (specifically 0.87, 0.66, and 0.44 wt %), was also evaluated. The additive was totally and easily soluble in biodiesel just under stirring. The oxidation stability of these biodiesel samples doped with BHT was measured with the same equipment and following the same procedure as with the bio-oil antioxidant additives.

## 3 Results and Discussion

First, this section presents how the mass of bio-oil is distributed between the different fractions resulting from the fractionation procedure. Then, the focus is on the characterization results, which are grouped according to the analytical technique for clarity. Discussion of each technique’s results includes the comparison of the bio-oil fractions separated first by solubility (in water and DCM) and the subfractions isolated then by molecular size. Finally, data of AntiOxP parameter are presented and discussed according to the chemical characterization of bio-oil fractions.

### 3.1 Mass Distribution after Bio-oil Fractionation

#### 3.1.1 Bio-oil Fractionation by Solvent Extraction

The mass yield of each bio-oil fraction obtained after solvent extraction is as follows: WI-DCMI: 15.5 wt %, WI-DCMS: 11.6 wt %, WS-DCMS: 23.5 wt % and WS-DCMI: 22.1 wt % (this latter one calculated by difference considering the other three fractions and the water content, 27.3 wt %). These values are similar to those obtained in other works that applied the original fractionation method to lignocellulosic bio-oil.^24,45^ Therefore, despite using a higher amount of bio-oil and only DCM as an organic extracting solvent after the water extraction, the results were comparable, representing an improvement in the simplicity of the methodology for solvent extraction fractionation.

#### 3.1.2 Fractionation of DCM-Soluble Fractions by Preparative-SEC

**Figure 1:**
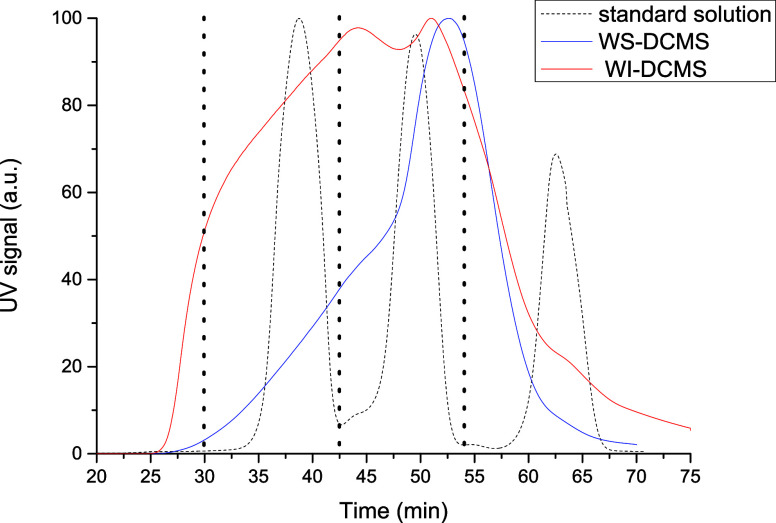
Preparative-SEC fractionation spectra: standard solution (dotted black line), WS-DCMS fraction of bio-oil (blue line) and WI-DCMS fraction of bio-oil (red line).

Both DCM-soluble fractions obtained from solvent fractionation of bio-oil (WI-DCMS and WS-DCMS) were subsequently separated by molecular size according to the elution time previously set with three compounds present in a standard solution (Figure 1). The elution profiles obtained for WS-DCMS and WI-DCMS (also shown in Figure 1) show an almost continuous elution signal, which means a wide range of molecular weights in each fraction. Therefore, although size fractionation of WI-DCMS and WS-DCMS is possible to some extent with preparative-SEC, narrow and clear peaks are not expected for the mass weight distribution of each subfraction, as it will be further confirmed by analytical SEC.

Four fractions were recovered from the WI-DCMS according to the elution time of the standard compounds: (i) a heavier fraction with elution times shorter than 30 min (WI-DCMS-H), (ii) a fraction in the size range of the tetramer used as standard, which eluted at times between 30 and 42.5 min (WI-DCMS-T), (iii) a fraction in the size range of the dimer used as standard, with elution times between 42.5 and 54 min (WI-DCMS-D) and (iv) a fraction in the size range of the monomer used as standard, eluting over 54 min (WI-DCMS-M). For the WS-DCMS, only three fractions were recovered, as the amount collected before 30 min was negligible compared to the total amount of the sample, thus showing a smaller presence of bigger molecules. These three fractions were named accordingly as WS-DCMS-T, WS-DCMS-D and WS-DCMS-M.

**Figure 2:**
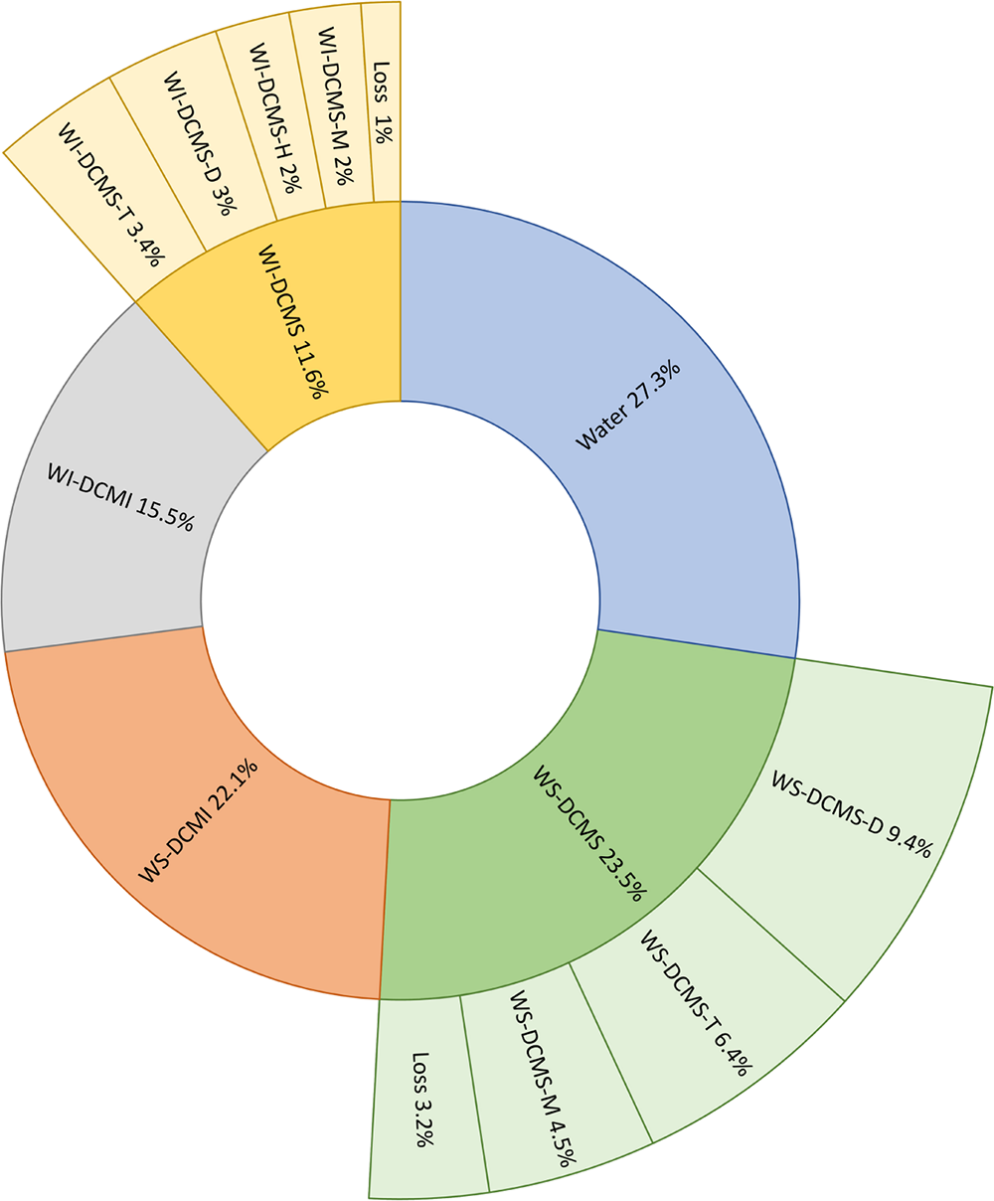
Mass distribution of bio-oil after solvent extraction and subsequent preparative-SEC fractionation.

Mass yields of the different subfractions separated by size from WS-DCMS were the following (mean ± standard deviation of at least three replicates): 27 ± 3 wt % of the injected sample was collected as WS-DCMS-T, 40 ± 9 wt % as WS-DCMS-D and 19 ± 5 wt % as WS-DCMS-M. For the WI-DCMS fraction, 17 ± 4 wt % of the injected fraction corresponded to WI-DCMS-H, 30 ± 6 wt % as WI-DCMS-T, 27 ± 1 wt % as WI-DCMS-D and 17 ± 3 wt % as WI-DCMS-M. The nonrecovered fraction of the injected sample (losses) was around 14 wt % for the WS-DCMS and 9 wt % for WI-DCMS. Figure 2 shows a schematic summary of the bio-oil mass distribution after the solvent extraction and the size fractionation. WS-DCMS-D was the most abundant subfraction collected after the complete fractionation procedure and roughly represented 10 wt % of the initial bio-oil.

### 3.2 Chemical Characterization of Bio-oil Fractions

#### 3.2.1 Synchronous UV-Fluorescence: Size Characterization

**Figure 3:**
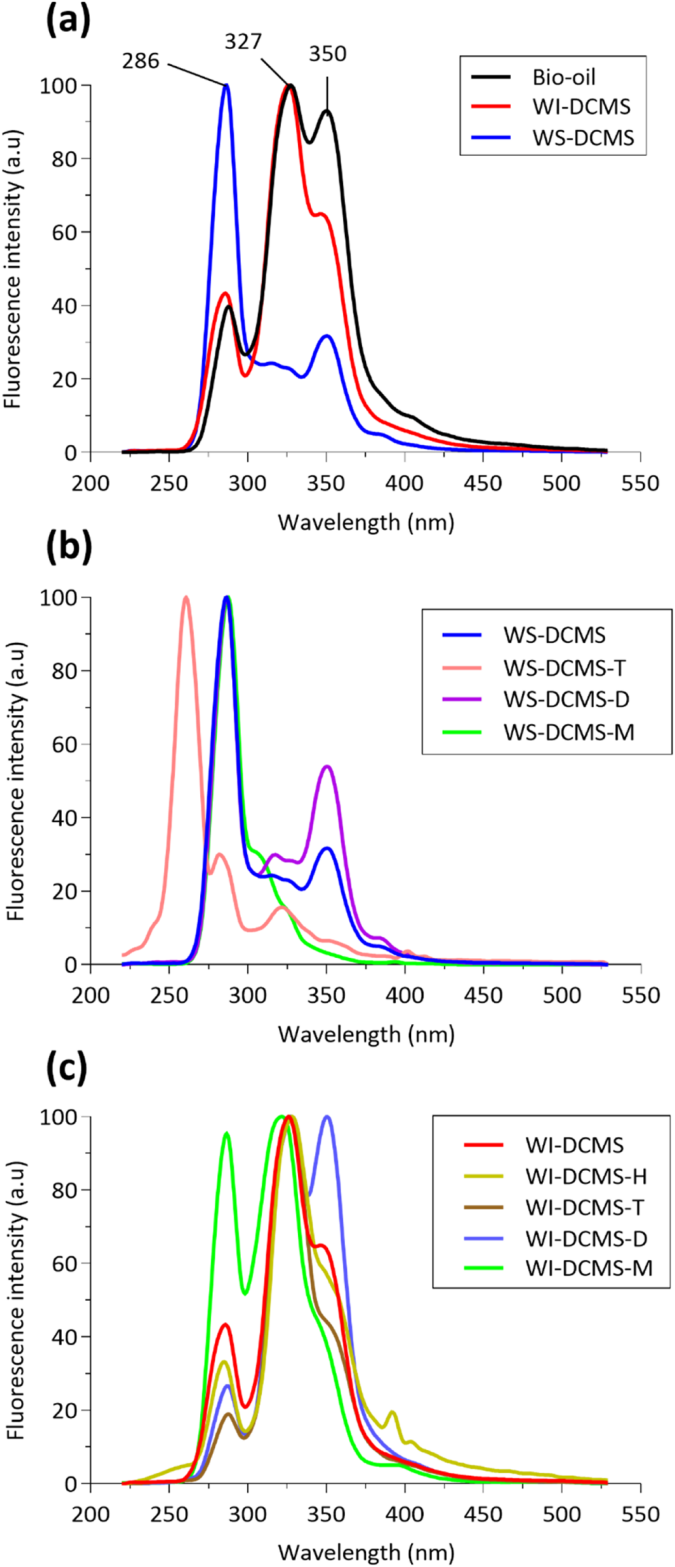
Fluorescence spectra of (a) bio-oil and fractions separated by solvent extraction, (b) WS-DCMS and its size-subfractions and (c) WI-DCMS and its size-subfractions.

The emission in synchronous UV-fluorescence is related to the number of conjugated bonds, as those expected between moieties with resonance connected by an oxygen linkage. Thus, this technique can be useful in distinguishing between phenol monomers and oligomers^42^ since phenolic oligomers are potentially more conjugated and emit at longer wavelengths. Figure 3 shows the normalized fluorescence spectra (the highest fluorescence emission in each sample was assigned to 100 arbitrary units) obtained after excitation with an offset of 20 nm for the whole bio-oil and its fractions.

Synchronous UV-fluorescence spectra showed that both the solvent extraction (Figure 3a) and the subsequent fractionation by molecular size (Figure 3b,c) have indeed provided separation of chemically different compounds, as the obtained fractions presented different spectra and, therefore, contain compounds with different resonant character, which can be attributed to different molecular size of phenolic compounds. Table 1 shows the area percentages resulting after integrating the spectra in the previously defined wavelength intervals. These area percentages can be qualitatively related to the abundance of aromatics with different sizes.

**Table 1:** Area distribution (%) as a Function of the Wavelength (λ) in the Fluorescence Spectra

**sample**	**percentage of area forλ < 300** nm	**percentage of area forλ = 300**	**percentage of area forλ > 340** nm
**bio-oil**	12.1	42.1	45.8
**WS-DCMS**	51.4	24.6	23.9
WS-DCMS-T	76.3	14.0	9.6
WS-DCMS-D	42.0	25.4	32.6
WS-DCMS-M	68.6	27.3	4.1
**WI-DCMS**	17.4	48.4	34.2
WI-DCMS-H	14.1	43.6	42.3
WI-DCMS-T	9.1	56.1	34.9
WI-DCMS-D	9.2	44.3	46.5
WI-DCMS-M	31.7	49.7	18.6

Results in Table 1 show that the percent area related to the heaviest compounds (λ > 340 nm) was higher in the starting bio-oil than in WI-DCMS and WS-DCMS, which could be explained by the presence of high molecular weight pyrolytic lignin in bio-oil, which would remain in WI-DCMI fraction after solvent extraction. The percentage of area related to the lightest compounds (λ < 300 nm) was much lower for the WI-DCMS fraction than for the WS-DCMS fraction, while the percent area related to emission in the λ = 300–340 nm interval was higher in WI-DCMS. Therefore, according to these results, it can be stated that WI-DCMS contains a higher ratio of heavier phenolic compounds, as will be later confirmed by the results of the SEC (Section 3.2.4).

After size fractionation of WS-DCMS, WS-DCMS-M and WS-DCMS-D followed the expected trend of shifting to larger areas at longer wavelengths as the sample was supposed to contain heavier compounds. Therefore, as only resonant molecules are observed with this technique because of their fluorescence, WS-DCMS-D is believed to contain bigger phenolics (aromatics) than WS-DCMS-M. On the other hand, the UV-fluorescence spectrum of the subfraction WS-DCMS-T was significantly different from the spectra of any of the other samples. First, it showed a maximum peak at around 255 nm instead of around 286 nm. Second, the area distribution was noticeably shifted to λ < 300 nm, pointing to compounds with less resonance. The interunit linkages such as resinol or phenylcoumarane in the oligomeric phenolic fractions prevent resonance, making the fluorescence nonquantitative. Moreover, compounds contributing to the high molecular weight of this T-subfraction could be different from phenolic oligomers, such as pyrolytic sugar oligomers.

The same behavior was observed with the size-subfractions separated by preparative-SEC from WI-DCMS: the monomeric subfraction (WI-DCMS-M) showed a higher percentage of the area related to λ < 300 nm than the subfraction WI-DCMS-D, while the heaviest subfractions (both WI-DCMS-T and WI-DCMS-H) did not exhibit higher percentages of area as the wavelength increased, pointing to the absence of resonance in their big structures.

#### 3.2.2 FTIR Spectroscopy: Identification of Functional Groups

**Figure 4:**
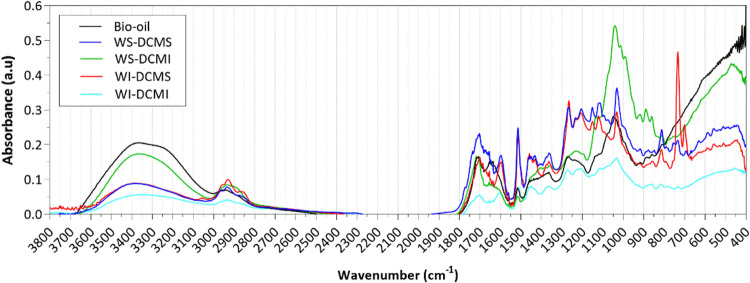
FTIR spectra of bio-oil and fractions obtained from the solvent fractionation procedure.

The absorbance FTIR spectra of the raw bio-oil and the fractions obtained by solvent fractionation are shown in Figure 4 and commented on according to the literature.^46,47^ The spectra of the two phases insoluble in DCM are also included for comparison purposes. As can be seen, the whole spectrum of the WI-DCMI presented less marked peaks than those obtained for the other three fractions and resembles char spectra. The wide band between 3600 and 3000 cm^–1^, attributed to OH stretch, was stronger for the raw bio-oil (high water content of 27.3 wt %) and the WS-DCMI fraction (containing anhydrosugars, low molecular weight acids and hydroxy acids). In the wavenumber interval 1740–1650 cm^–1^, marked peaks were observed in all samples pointing to carbonyls (C=O stretch) in unconjugated ketones and esters, and conjugated aldehydes and carboxylic acids from carbohydrate origin or p-substituted aryl ketones. Signals from 1605 to 1505 cm^–1^, usually attributed to the aromatic skeletal vibrations, were more intense in the two DCM-soluble fractions, which in turn points to a higher content of phenolic compounds with respect to the DCM-insoluble fractions. In the DCM-soluble fractions, higher-intensity peaks were observed at around 1515 cm^–1^ than at 1600 cm^–1^, pointing to a major presence of guaiacyl-units in comparison with syringyl-ones,^47^ which is typical from a softwood bio-oil (pine in this case); the ^31^P-NMR results also confirmed this observation (see Section 3.2.6). Around 1460 cm^–1^, a shoulder-shape peak assigned to C–H deformations in alkane (–CH_3_ and –CH_2_) is especially intense for the subfractions WS-DCMS and WI-DCMS, thus indicating a higher proportion of alkyl chains. Accordingly, in the fingerprint region, the two DCM-soluble fractions showed other similarities among them, for example, in the peaks around 1265 cm^–1^, related to guaiacyl-units plus C=O stretch (the high content of vanillin in DCM-soluble fractions observed in the GC-MS/FID analyses could explain it, Section 3.2.3). The marked peak at 1030 cm^–1^ in the fingerprint region of the WS-DCMI faction could be attributed to the C–O–C bond present in anhydrosugars such as levoglucosan or furans like 2,5-dimethylfuran or maltol (see spectra of levoglucosan furan, 2,5-dimethyl and maltol in the NIST Chemistry Webbook database). Moreover, the WI-DCMS fraction showed a well-defined peak at 730 cm^–1^, which was not present in the other fractions and could be attributed to C=C bending in fatty acid chains.^46^

**Figure 5:**
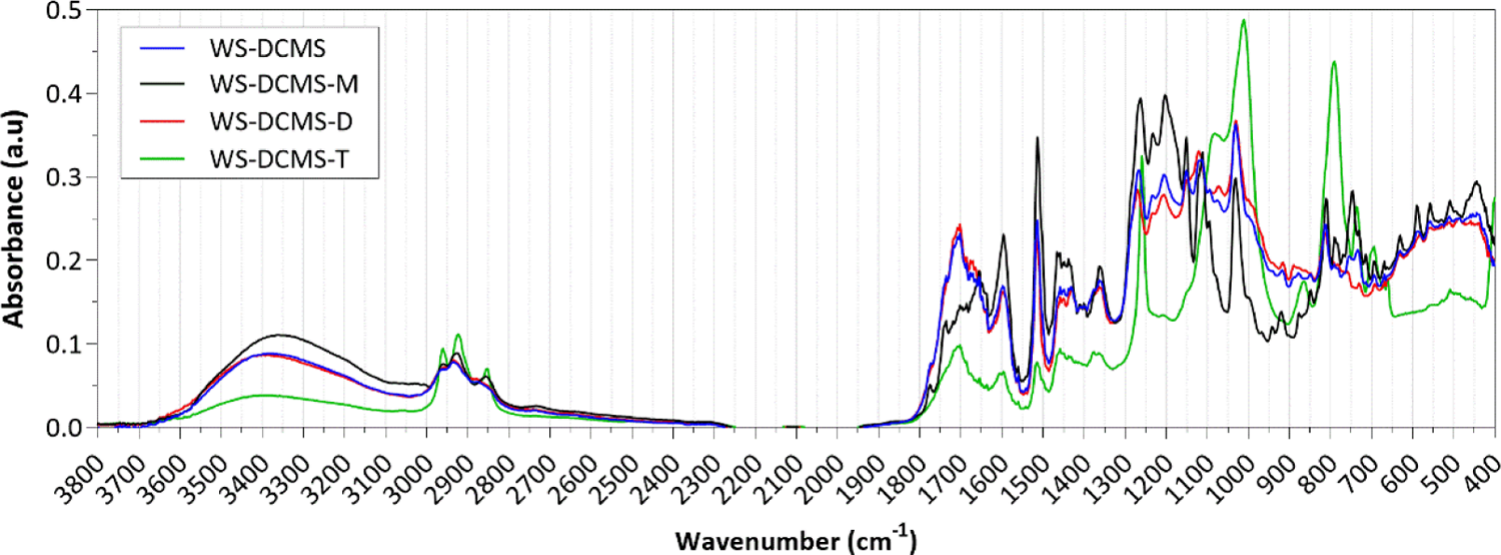
FTIR spectra of the subfractions obtained by preparative-SEC from the WS-DCMS.

Figure 5 shows the FTIR spectra corresponding to the size-subfractions obtained from WS-DCMS. As can be seen, the shape of the FTIR spectrum of the WS-DCMS-D subfraction was the most similar to that of the parent fraction before molecular size separation (WS-DCMS), likely because of its highest mass fraction in the WS-DCMS fraction. The spectra of both samples showed a similar and intense band at 1740 and 1650 cm^–1^, pointing to the highest presence of carbonyl groups; this result was confirmed by the quantitative ^13^C-NMR (see Table 3 in Section 3.2.5). The spectra of the subfractions WS-DCMS-M and WS-DCMS-T showed noticeable differences. The monomer-rich fraction presented a wider and more intense band between 3600 and 3100 cm^–1^, indicating a higher presence of −OH bonds in this subfraction. Peaks at 1515 and 1265 cm^–1^, related to aromatic skeletal vibrations (specifically to guaiacyl-units and guaiacyl plus C=O stretch, respectively), were more intense in this WS-DCMS-M subfraction. In the heaviest subfraction (WS-DCMS-T), two peaks around 1010 and 790 cm^–1^, owing to C–H and C=C bending, stood out from the rest of the subfractions. In addition, this fraction was also characterized by remarkable peaks at 3000–2800 cm^–1^ (C–H stretch), indicating the presence of alkyl chains.

**Figure 6:**
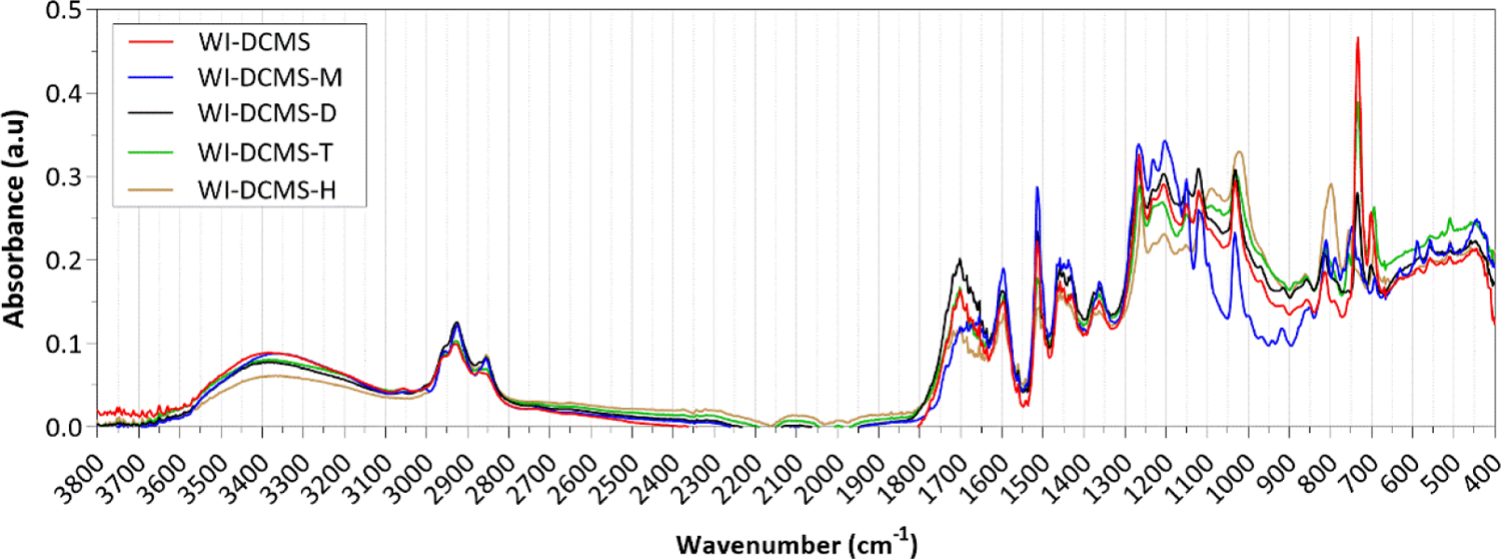
FTIR spectra of the subfractions obtained by preparative-SEC from the WI-DCMS.

The FTIR spectra of the size-subfractions separated from the WI-DCMS are shown in Figure 6. In this case, the spectra of the different subfractions presented less markable differences. The high concentration of aromatic rings in the WI-DCMS-M subfraction, specifically methoxy phenols, also confirmed by the GC-MS/FID results (see Section 3.2.3), was reflected by the intense peaks at 1515 and 1265 cm^–1^ corresponding to guaiacyl-units and guaicyl-units plus C=O. As for the WS-DCMS subfractions, the presence of carbonyl bonds (band around 1700 cm^–1^) was higher for the subfraction eluted in the dimer range (WI-DCMS-D), which could be attributed to a high concentration of fatty acids and fatty acid methyl esters, as observed in the GC-MS/FID results.

#### 3.2.3 Gas Chromatography: Characterization of the Volatile Fraction

**Figure 7:**
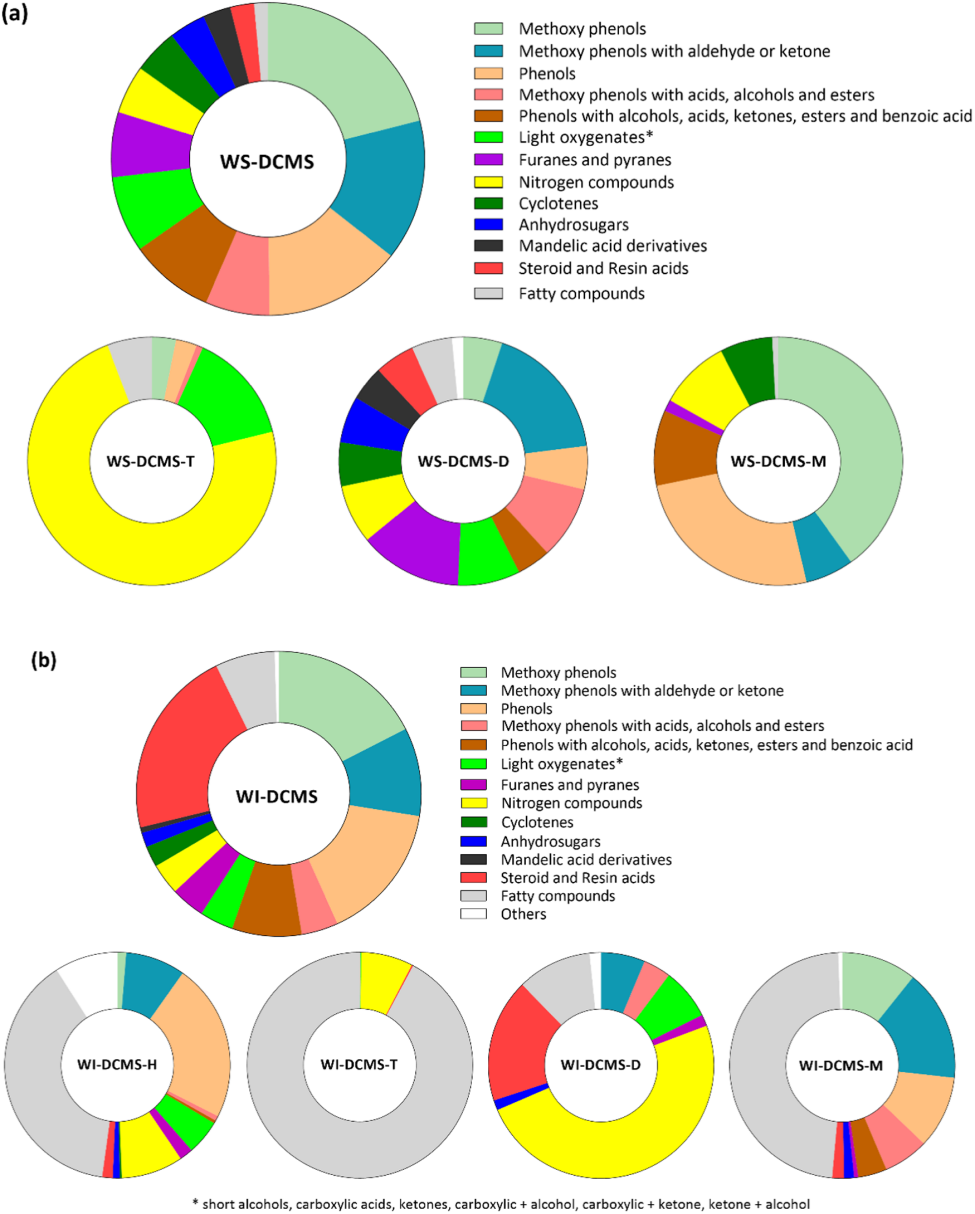
Mass percentage distribution of GC-elutable chemical families in (a) WS-DCMS and (b) WI-DCMS and size-subfractions separated from both.

Figure 7 shows the mass percentage distribution of the GC-elutable compounds grouped by chemical families in the DCM-soluble fractions and size-subfractions separated by preparative-SEC. Table SI-2 in the Supporting Information shows the detailed list of compounds identified and quantified by GC-MS/FID in the different fractions and subfractions. Concerning the WS-DCMS fraction (Figure 7a), the main families of compounds detected were: (i) methoxy phenols (∼21%), including methyl guaiacol, guaiacol, eugenol and syringol among other compounds; (ii) methoxy phenols with an aldehyde or ketone side chains (∼15%), including vanillin, acetovanillone and coniferyl aldehyde; (iii) phenols (∼14%), including catechol, phenol and short chain alkyl phenols; (iv) phenols with side chains containing acids, alcohols and esters (∼9%), including 2-hydroxyphenethyl alcohol, p-coumaric alcohol or salicylic acid, among others, and, last (v) methoxy phenols with side chains containing acids, alcohols, and esters (∼7%), including 3-vanilpropanol and vanillic acid. Therefore, compounds whose structure contains a phenol ring represent around 65% of this fraction. Apart from these phenol-derived compounds, other chemical families like furans and pyrans (∼7%), such as 3-methyl-2-furoic acid, maltol or 2(5H)-furanone 3-methyl-, light oxygenates (∼8%), such as ethylene glycol or 2-ketobutyric acid, and nitrogen compounds (∼5%), such as acetamide or uracil, were detected in WS-DCMS fraction. Much lower concentrations of cyclotenes, anhydrosugars, mandelic, steroids, resin acids and fatty compounds were also identified.

Regarding the size-subfractions separated from WS-DCMS by preparative-SEC, the WS-DCMS-M was mainly composed of methoxy phenols (∼40%), phenols (∼25%), phenols with side chains containing acids, alcohols and esters (∼10%) and methoxy phenols with an aldehyde or ketone side chains (∼6%), in addition to nitrogen compounds (∼9%) and cyclotenes (∼7%). Therefore, this subfraction was enriched in phenols and methoxy phenols compared to the parent WS-DCMS fraction (82 vs 65%). Unlike it, WS-DCMS-D showed lower content in phenolic compounds (41%) than the parent fraction (65%). Considering the relatively low molecular weight of the compounds present in fractions WS-DCMS-M and WS-DCMS-D (see SEC and DOSY-NMR results in Section 3.2.4), they are expected to be virtually GC-elutable, so given percentages can be directly understood as final mass concentrations. Although the separation of compounds in the fractions WS-DCMS-M and WS-DCMS-D was not perfect, and some of the compounds appeared in both subfractions, most of them have been significantly concentrated in one of these two subfractions. In this way, the major compounds in the WS-DCMS-M fraction were 2-methoxy-5-methylphenol (22.5 wt %), guaiacol (10.1 wt %), catechol (7.1 wt %) and vanillin (5.5 wt %), while the most abundant compounds in WS-DCMS-D were vanillin, coniferyl aldehyde, acetovanillone, 3-methyl-2-furanoic acid and 3-vanilpropanol, showing all of them concentrations around 5 wt %. Only vanillin was quantified in both fractions at significant concentrations among these compounds. Regarding the last size-subfraction (WS-DCMS-T) and considering the results of the SEC and DOSY-NMR analyses, it can be stated that only a small portion of WS-DCMS-T will be GC-elutable. Nitrogen-containing compounds such as *N*-methylpropionamide and ethanolamine were especially abundant among the identified compounds.

Concerning WI-DCMS (Figure 7b), different families of compounds were also identified. Lower percentages of phenolic monomers were observed concerning WS-DCMS (55% in WI-DCMS vs 65% in WS-DCMS), while more steroid and resin acids (22% in WI-DCMS vs 2.5% in WS-DCMS) and more fatty compounds (7% in WI-DCMS vs 2% in WS-DCMS) were detected. On the other hand, the presence of nitrogen compounds (3.5%) and anhydrosugars (1.7%) was slightly lower in WI-DCMS than in WS-DCMS. Meanwhile, noticeable differences in the composition of each group could be observed for the different subfractions obtained after size fractionation. Molecular size separation of this fraction did not achieve the concentration of the phenolic compounds in any of its subfractions, as happened with WS-DCMS-M coming from WS-DCMS. Important percentages of some fatty compounds, like 9,12-octadecadienoic acid methyl ester, 9-octadecenoic acid methyl ester and hexadecenoic acid methyl ester, were found in all the separated size-subfractions, which points to a bad separation of these compounds with the employed resin (Bio-Bead S-X3). The important presence of fatty compounds (especially methyl esters) agrees with the remarkable peak showed at wavenumber 1700 cm^–1^ in the FTIR spectra of WI-DCMS fraction and its size-subfractions, as well as with the results of the ^13^C-NMR, which showed peaks in the interval 20–50 ppm (typical of fatty compounds).

#### 3.2.4 Analytical SEC and DOSY-NMR: Size Characterization

**Figure 8:**
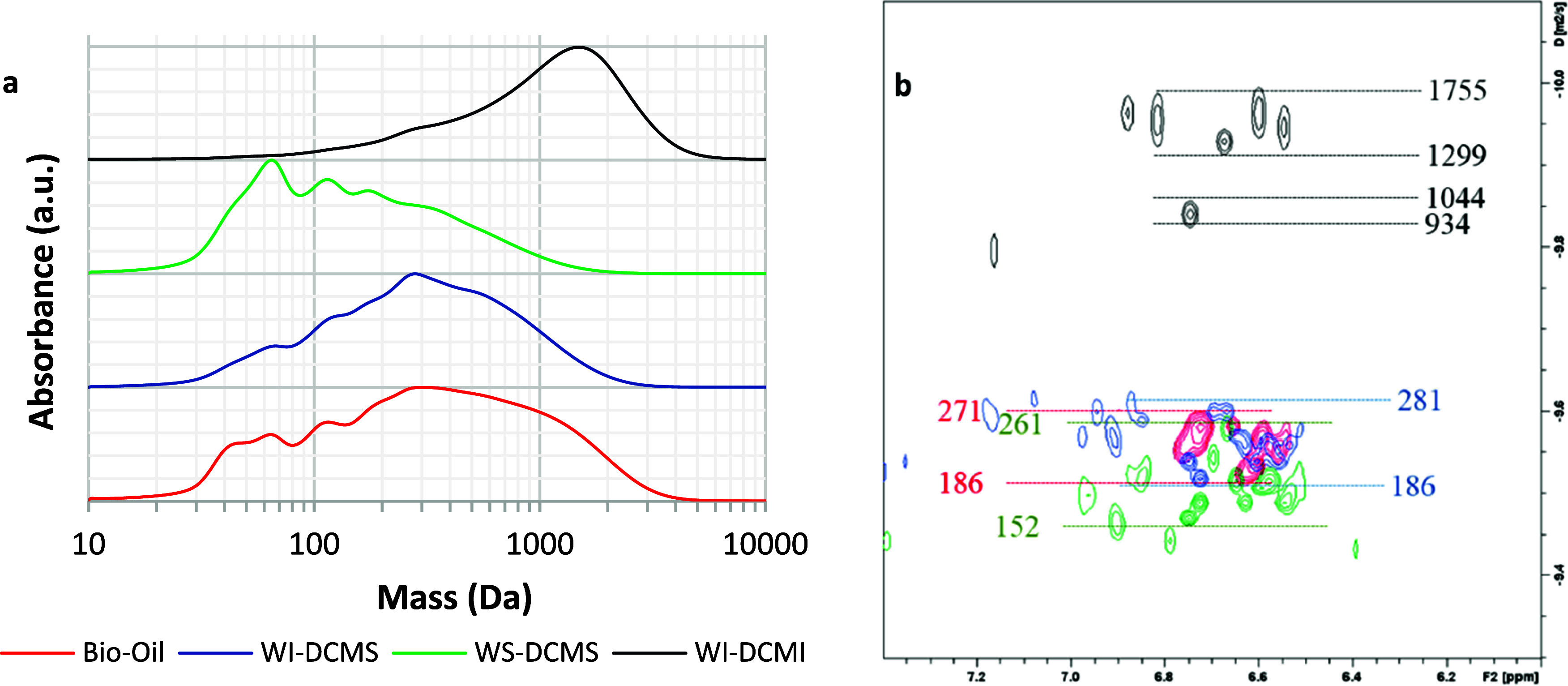
(a) SEC chromatograms (UV detector at 254 nm) and (b) DOSY spectra in the aromatic region for bio-oil (red traces), WI-DCMS (purple traces), WS-DCMS (green traces) and WI-DCMI (black traces). (*Red, green and blue figures correspond to estimated mass using PEGP calibration, and black figures correspond to PS calibration)*.

Analytical SEC and DOSY-NMR techniques were applied to estimate the molecular weight distribution of the samples. Bio-oil and samples arising from solvent fractionation were examined by SEC using a UV absorbance detector (SEC-UV) at 254 nm, allowing the detection of aromatics (Figure 8a). As can be observed, the mass distribution noticeably changed when comparing the original bio-oil to the fractions obtained after solvent fractionation (WI-DCMS, WS-DCMS and also WI-DCMI). The mass distribution curve of WI-DCMI was shifted to higher molecular masses than the starting bio-oil sample (Figure 8a), with its maximum at 1638 Da (*M*_w_ = 1897 Da, *M*_n_ = 1280 Da, Table 2), which is noticeably higher than other values reported in the literature for pyrolytic lignin from pine (*M*_w_ = 690 Da), considering pyrolytic lignin as the water-insoluble fraction of bio-oil, without any other extraction step.^48^ On the other hand, the mass distribution curve of WS-DCMS was shifted to lower values compared to starting bio-oil, reaching its maximum at 70 Da (*M*_w_ = 475 Da and *M*_n_ = 216 Da, Table 2). These results evidence that solvent fractionation already provided a first-size fractionation. The mass distribution for WI-DCMS was closer to that reported for the original bio-oil, reaching its maximum at *ca*. 300 Da (*M*_w_ = 763 Da and *M*_n_ = 418 Da, Table 2).

**Table 2:** Estimation of Molecular Weight of Bio-oil and Its Fractions According to Analytical SEC and DOSY-NMR *(Molecular Weight Estimated Using PS and PEGP Calibration*)

	SEC (λ = 254 nm)			DOSY-NMR	
	*M*_w_ (Da)	*M*_n_ (Da)	*n*	mass interval (PS calibration)	mass interval (PEGP calibration)
**bio-oil**	1223	541	2.26	291–408	186–271
**WS-DCMS**	475	216	2.20	245–396	154–262
WS-DCMS-T	990	653	1.52	867–1775	626–1375
WS-DCMS-D	462	266	1.74	252–417	158–277
WS-DCMS-M	235	85	2.78	197–282	120–179
**WI-DCMS**	763	418	1.83	291–422	186–281
WI-DCMS-H	1803	1218	1.48	not determined	not determined
WI-DCMS-T	1542	1079	1.43	1025–2209	755–1777
WI-DCMS-D	1015	387	2.62	297–689	190–461
WI-DCMS-M	299	147	2.04	168–297	101–190
WI-DCMI	1897	1280	1.48	934–1755	681–1375

DOSY-NMR spectroscopy (Figure 8b) confirmed the same observations when the aromatic region of the spectra was analyzed. Bio-oil and WI-DCMS samples presented very similar diffusion coefficients in the aromatic region, and the estimated masses were in the range of 186–281 Da according to the PEGP calibration performed. Lower masses were found in WS-DCMS at an estimated range of 154–262 Da, while the WI-DCMI fraction presented a higher apparent mass than the original bio-oil (681–1375 Da). Therefore, it can be assumed that most of the aromatic compounds with lower masses were transferred to the WS-DCMS fraction (the same finding was observed by UV-fluorescence spectroscopy), whereas oligophenols were mostly transferred to WI-DCMI.

The SEC chromatograms using a RID detector (Figure SI-2a in the Supporting Information) were used to estimate the molecular weight of compounds that did not present absorption at 254 nm, as those derived from saccharides. SEC analysis confirmed that the WS-DCMS fraction was shifted toward lower masses than WI-DCMS (and bio-oil), which, besides the presence of monomeric phenolic compounds, could be attributed to the presence of some sugars or anhydrosugars that were not detected by UV absorption. A careful analysis of the DOSY spectra (Figure SI-2b in the Supporting Information) evidenced signals in the 4.5–5.5 ppm range with high diffusion coefficients, potentially corresponding to anomeric carbon and hydroxy groups from saccharides or anhydrosaccharides with low molecular weight.^38^ This finding has been further confirmed by HSQC spectroscopy: WS-DCMS presented noticeable contours in the 4.5–5.5 ppm/95.0–105.0 ppm ranges (Figure SI-3 in the Supporting Information), corresponding to anomeric carbon atoms.^49^ Although with lower intensity, similar signals were observed in the WI-DCMS fraction.

**Figure 9:**
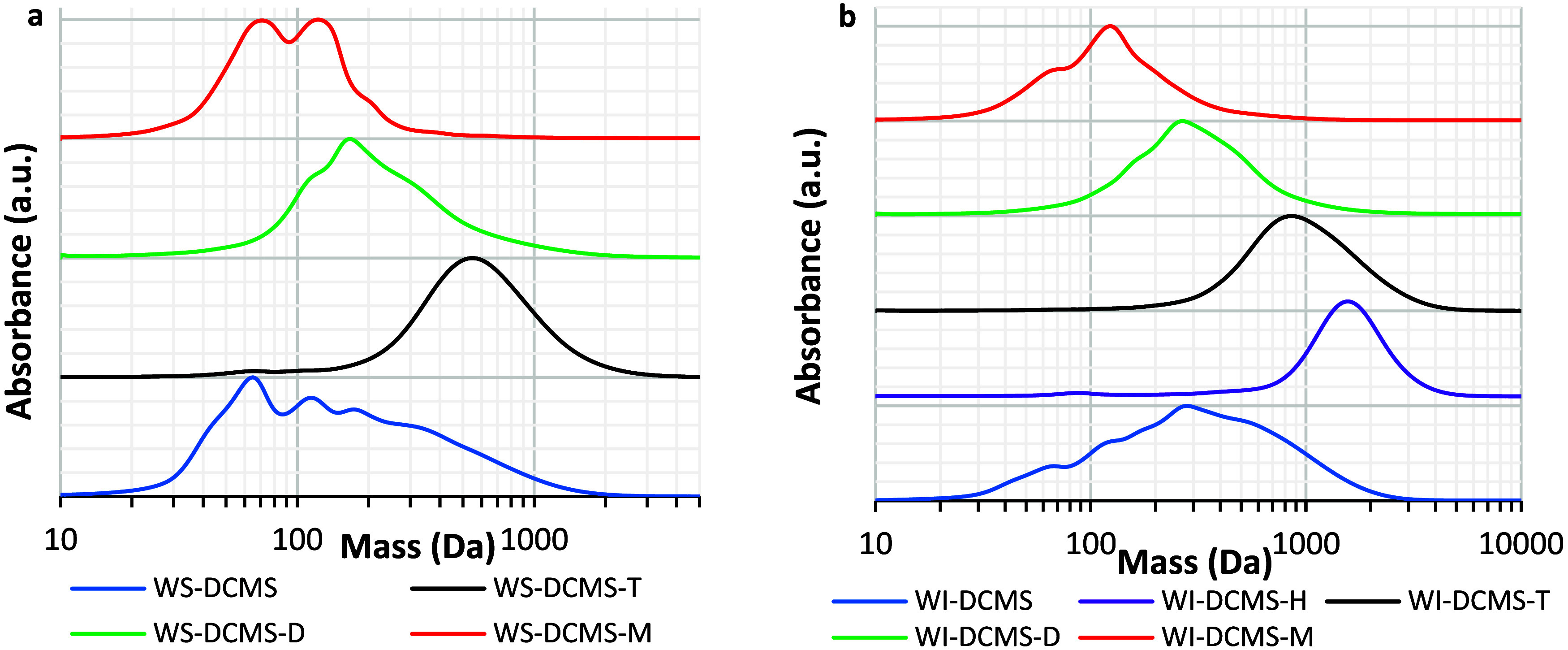
SEC chromatograms (λ=254 nm) of bio-oil subfractions separated by preparative-SEC fractionation of (a) WS-DCMS and (b) WI-DCMS.

SEC analysis of size-subfractions (Figure 9) confirmed the efficacy in fractionating WS-DCMS and WI-DCMS by preparative-SEC. In both cases, the H-, T-, D- and M-subfractions showed the expected trend in their average molecular weight. The SEC chromatograms for the WS-DCMS size-subfractions (Figure 9a) demonstrated a good separation with maxima at 556 Da for WS-DCMS-T, 173 Da for WS-DCMS-D and a bimodal distribution, at 122 and 73 Da, for the WS-DCMS-M (average *M*_n_ values of 653 Da, 266 and 85 Da, respectively, see Table 2). The fractionation obtained for WI-DCMS was even better (Figure 9b) with maxima at 1621 Da, 877 Da, 277 and 123 Da for H-, T-, D- and M-subfractions, respectively, which is in good agreement with the corresponding values of *M*_n_ (1218 Da, 1079 Da, 387 and 147 Da respectively, see Table 2).

**Figure 10:**
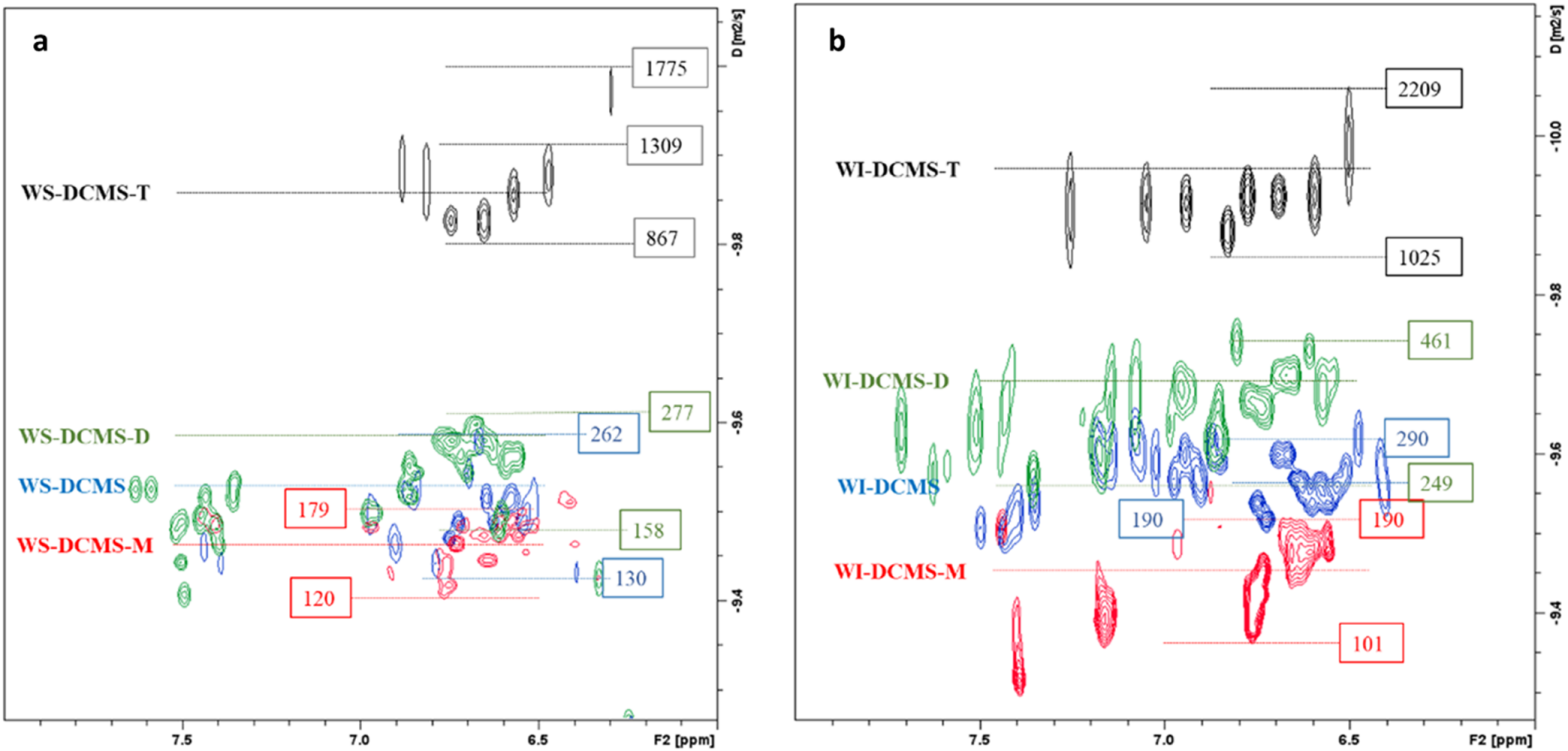
2D-DOSY spectra of subfractions after size fractionation of (a) WS-DCMS and (b) WI-DCMS. *(Red, blue and green figures correspond to PEGP calibration, and black figures correspond to PE calibration)*.

The DOSY spectra of the size-subfractions separated by preparative-SEC (Figure 10) also allowed the estimation of their molecular weight. Thus, in the WS-DCMS subfractions, the apparent masses were in the range of 867–1775, 158–277, and 120–179 Da for T-, D- and M-subfractions, respectively. As expected, higher apparent masses were estimated for the WI-DCMS fractions: 1025–2209, 190–461, and 101–190 Da for T-, D- and M-subfractions, respectively (the H-subfraction was not measured due to the low amount isolated). Therefore, the estimated masses using SEC and DOSY-NMR were in good agreement. Noteworthy, the low intensity for the aromatic region (6.0–8.0 ppm) in both T-subfractions suggests a low presence of phenolic structures in these heavier subfractions.

#### 3.2.5 ^13^C-NMR Spectroscopy: Distribution of C Atoms in Different Functional Groups

The fractions WS-DCMS and WI-DCMS and their size-subfractions separated by preparative-SEC were analyzed using quantitative ^13^C-NMR spectroscopy. The percentage for each type of C signal was calculated by integrating the spectra (Table 3; chemical shifts adapted from literature).^30,50^ The resulting spectra are shown in the Supporting Information (Figure SI-5).

**Table 3:** Distribution of C Atoms in Different Functional Groups in Bio-oil and Its Separated Fractions

	**percent of signal per type of C atom**												
functional group	aldehydes	ketones	carboxylic acids	total carbonyl	C(aromatic)–O	C(aromatic)–C	C(aromatic)–H	total aromatic	anomeric C	sugars/aliphatic C–O	total sugars	O–CH_3_	aliphatic
ppm	^>200^	180–200	166.5–180		142.0–166.5	125.0–142.0	105–125		90–110	60–90		50–60	0–50
bio-oil	4	0	6	10	7	0	9	16	7	35	42	6	17
WS-DCMS	2	1	4	7	18	6	24	48	1	11	12	9	20
WI-DCMS	1	1	2	4	17	8	25	50	2	5	7	20	15
WS-DCMI	3	0	4	7	1	0	2	3	20	63	83	3	5
WS-DCMS-T	0	0	0	0	0	0	0	0	0	0	0	38	62
WS-DCMS-D	1	2	4	7	18	7	22	47	2	12	14	13	19
WS-DCMS-M	0	1	1	2	19	5	36	60	0	6	6	10	14
WI-DCMS-T	0	0	5	5	3	4	1	8	0	0	0	11	76
WI-DCMS-D	0	1	3	4	13	9	6	28	0	0	0	16	52
WI-DCMS-M	0	0	2	2	9	8	21	38	0	1	1	15	37

One of the most remarkable findings after solvent fractionation is related to the percent of C atoms contained in carboxylic acids: bio-oil presented the highest signal percent (6%), while this percentage decreased to 4% in WS-DCMS and 2% in WI-DCMS, suggesting that none of these fractions was enriched in such chemical functionality, and neither WS-DCMI, whose signal for carboxylic acids was 4%. On the other hand, the cumulated percent signals for sugars or aliphatic C–O decreased from 42% in bio-oil to 12% in WS-DCMS and 7% in WI-DCMS, whereas in the case of WS-DCMI raised to 83% of the C atoms, suggesting that most of this fraction corresponded to saccharides or derived compounds. Regarding C atoms taking part in aromatic rings, the total percent was noticeably higher either in WI-DCMS or WS-DCMS (*ca*. 50% in both cases) than in bio-oil (16%), which confirms that DCM efficiently concentrated aromatic structures upon the solvent fractionation used in this work.

The results from ^13^C-NMR confirmed that, besides leading to bio-oil subfractions different in molecular sizes, preparative-SEC fractionation also provided differences in chemical functionalities (Table 3). Almost no aromatic C signal could be detected in the T-subfractions (neither in WS-DCMS-T nor in WI-DCMS-T), in which most of the C signals corresponded to aliphatic C atoms; this presence of aliphatic chains was already observed in the GC-detectable portion of the samples (Figure 7). This is also in good agreement with the low intensity presented in DOSY spectra for the aromatic-hydrogen atoms and the presence, in both cases, of alkyl hydrogen atoms at lower diffusion coefficients than those of the aromatic regions, which evidence that aromatic and alkyl hydrogen atoms are not part of the same molecule. Hence, according to their chemical shifts, alkyl hydrogen atoms may belong to long-chain aliphatic ketones, esters, or carboxylic acids, which may be produced from fatty components or sugar degradation in the pyrolysis process.

Size fractionation of WS-DCMS led to a very effective separation of the compounds bearing aliphatic alcohols, which could be attributed to anhydrosugars. The content of this type of C atoms (anomeric C atoms in the 90–105 ppm range) in WS-DCMS was 12% of the C signals, which was maintained around 14% in WS-DCMS-D and reduced to 6% in WS-DCMS-M. This suggests that sugar derivatives present in the WS-DCMS fraction were significantly eluted in the range of the aromatic dimeric compounds (D-subfraction). Very low-intensity signals were detected in the 60–105 ppm range (typical for both aliphatic polyols and saccharide compounds) in the WS-DCMS-M sample, while these signals were not detected in WS-DCMS-T. On the other hand, the content of aromatic C atoms was noticeably higher in WS-DCMS-M (60%) than in WS-DCMS-D (47%) or even in the parent solution WS-DCMS, which points to an important concentration of aromatic structures in these two subfractions, especially in the monomer-rich one. Finally, as for WS-DCMS-T, ^13^C signals corresponding to aromatic C were not detected, and only aliphatic and methoxy signals were observed that could be consistent with long-chain acid esters.

Concerning WI-DCMS, both D- and M-subfractions presented similar percentages of C atoms taking part in carboxylic groups (*ca*. 2%) and sugar/aliphatic C–O (*ca*. 1%). The aliphatic content was more important in WI-DCMS-D than in WI-DCMS-M (52 vs 37%). As in the case of WS-DCMS subfractions, an increase in the percent of aromatic carbons was observed as reducing the molecular weight of the subfractions (8, 28, and 38% for T-, D- and M- subfractions, respectively). This indicates that most of the aromatic moieties were transferred to M-subfraction, which, according to SEC and DOSY-NMR, are monomeric.

NMR analysis has been useful to compare with GC-MS results, as GC-MS analysis should be carefully considered as not all the compounds present in each bio-oil fraction can be detected by GC. GC-MS is only expected to provide the quantitative composition of volatile fractions. However, given the wide variety of compounds in bio-oil, other techniques are required to characterize bio-oil in depth.

The qualitative composition of each phase characterized by NMR points in the same direction as the GC results regarding the type of chemical families.

Thus, WI-DCMS-T and WS-DCMS-T fractions presented low amounts of aromatic compounds according to GC analysis, and so, quantitative ^13^C-NMR presented very weak signals in the aromatic region, as well as low contents of aromatic hydroxy groups, Ar–OH, according to ^31^P-NMR of the derivatized samples (see Section 3.2.6). On the other hand, WI-DCMS-T was rich in fatty compounds according to GC-MS, and the main ^13^C signals in the NMR spectrum were also detected in the aliphatic region (10–40 ppm).

Similarly, in WI-DCMS-D, most of the composition according to GC-MS corresponded to nitrogen-containing compounds and fatty compounds, whose signals are again found in the aliphatic region of the ^13^C spectrum. As for WS-DCMS-D, GC-MS presented a considerable amount of steroid and resin acid, mandelic acid derivatives, cyclotenes and phenyl and guaiacyl derivatives, which is consistent not only with the signals found in the ^13^C-NMR, but also with the estimated values for the aromatic hydroxy groups by ^31^P-NMR of the derivatized samples (see Section 3.2.6).

Finally, in WI-DCMS-M, 48 wt % of GC-detectable compounds were fatty compounds and 47% were phenyl and guaiacyl derivatives. This is again consistent with the integration of the quantitative ^13^C-NMR spectrum that showed that 37% of the C signal corresponded to alkyl carbon atoms, 2% to carboxy carbon atoms, whereas 38% corresponded to aromatic C atoms (Table 4). ^31^P-NMR of this derivatized sample showed an aromatic hydroxy content of 3.14 mmol Ar–OH/g (Section 3.2.6). Similarly, in WS-DCMS-M, 82 wt % of GC-detectable compounds corresponded to phenyl compounds, whereas 9% corresponded to amines and 6% to cyclotenes, which is again consistent with the quantitative ^13^C-NMR that showed that 60% of the signal corresponded to aromatic C atoms. In contrast, only 14% corresponded to alkyl C atoms. ^31^P-NMR of the derivatized WS-DCMS-M sample suggested an aromatic hydroxy content of 3.96 mmol Ar–OH/g (see Section 3.2.6), the highest among the analyzed samples.

**Table 4:** Antioxidant Effect of Bio-oil Fractions on Biodiesel Oxidation Stability

**sample** (number of replicates)	**ASD-%**	***t***_neat BD_**(min)**	***t***_doped BD_**(min)**	AntiOxP
**bio-oil** (4)	0.5 ± 0.1	24.1 ± 0.9	32 ± 2	16.7 ± 0.6
**WS-DCMS** (2)	0.54 ± 0.03	22.7	50 ± 2	50.0 ± 0.2
WS-DCMS-T (2)	0.46 ± 0.03	22 ± 1	25 ± 2	7.7 ± 0.4
WS-DCMS-D (3)	0.49 ± 0.02	20.8 ± 0.2	41 ± 2	41 ± 2
WS-DCMS-M (3)	0.44 ± 0.03	22.0 ± 0.8	59 ± 9	82 ± 15
**WI-DCMS** (2)	0.54 ± 0.04	20.7	49 ± 3	52 ± 2
WI-DCMS-H (3)	0.79 ± 0.08	21.2 ± 0.9	28 ± 2	8 ± 1
WI-DCMS-T (3)	0.5 ± 0.2	22.0 ± 0.3	33 ± 6	22 ± 6
WI-DCMS-D (2)	0.43 ± 0.01	22 ± 1	40 ± 2	41 ± 5
WI-DCMS-M (3)	0.44 ± 0.06	21.3 ± 0.5	34.4 ± 0.5	30 ± 6
**WI-DCMI** (2)	0.2 ± 0.1	21.4 ± 1.0	32.1 ± 6.1	51.1 ± 0.8

Therefore, it can be stated that, even if it is known that GC-MS cannot provide a full quantitative composition of heavy bio-oil fractions, the qualitative results concerning the type of chemical families are consistent with the NMR analyses, which do reflect all the compounds present in the samples, regardless of their volatility/molecular size.

#### 3.2.6 ^31^P-NMR Spectroscopy: Hydroxy Group Titration

^31^P-NMR spectroscopy after derivatization with 2-Chloro-4,4,5,5-tetramethyl-1,3,2-dioxaphospholane (TMDP), allowed the quantification of the hydroxy groups that were present in the bio-oil samples (Table SI-4 in the Supporting Information). The content of aromatic hydroxy groups in the raw bio-oil was 1.18 mmol/g, accompanied by 2.77 mmol/g of aliphatic hydroxy groups. This value must be carefully considered, as water, which also interferes in derivatization, was present in high content in the bio-oil sample (27.3 wt %).

As a result of solvent fractionation, the aromatic hydroxy group content increased to 2.72 mmol/g in WS-DCMS and 2.92 mmol/g in WI-DCMS. The WI-DCMI fraction showed the highest content of the aromatic hydroxy group, 3.54 mmol/g. Moreover, the results obtained from ^31^P-NMR spectroscopy highlighted that the preparative-SEC effectively increased the hydroxy group’s concentration in some specific fractions. The aromatic hydroxy content was 2.43 mmol/g in WS-DCMS-D and raised to 3.96 mmol/g in WS-DCMS-M. Similarly, the content of the aromatic hydroxy group was higher in WI-DCMS-D (3.96 mmol/g) and in WI-DCMS-M (3.14 mmol/g) than in the parent fraction WI-DCMS (2.92 mmol/g). As expected according to previous results, the content of the aromatic hydroxy group in T-subfractions was, in both cases, much lower (0.91 mmol/g for WS-DCMS-T and 1.72 mmol/g for WI-DCMS-T), which is consistent with the low percent of C signals in the 142.0–166.5 ppm range that correspond to aromatic C atoms forming C–O bonds (Table 3) and with the relatively low intensity of the DOSY contours in the aromatic region (Figure 10).

### 3.3 Antioxidant Potential of Bio-oil Fractions

The oxidation stability parameter, AntiOxP, previously defined in eq 2, is shown in Table 4 for the blends of biodiesel with (i) the whole bio-oil, (ii) the fractions separated by solvent fractionation (except for the fraction WS-DCMI, which was not tested as it mainly contains polar compounds like anhydrosugars that are poorly soluble in biodiesel and are out of the scope of this study), and (iii) the seven size-subfractions obtained by preparative-SEC (three from the WS-DCMS fraction and four from the WI-DCMS one).

Data in Table 4 highlight a significant scatter in the solubility of bio-oil in biodiesel, which can be attributed to the heterogeneous nature of this liquid. Scatter in the solubilized dosage was significantly reduced when working with bio-oil subfractions. As could be expected from chemical characterization, DCM-soluble fractions solubilized better in biodiesel (*ca*. 55 wt %) than the WI-DCMI fraction. After preparative-SEC fractionation, most of the subfractions solubilized in biodiesel around 45–50 wt % of the initial dosage (which was 1 wt %); only the heaviest fraction obtained from WI-DCMS (WI-DCMS-H) was significantly more soluble because of its highly aliphatic character.

Regarding the antioxidant performance, all bio-oil samples somewhat improved the biodiesel oxidation stability. Longer oxidation stability times were measured when incorporating WI-DCMS or WS-DCMS into biodiesel instead of the whole bio-oil (Table 4). Both fractions reached the same soluble dosage in biodiesel, leading to similar results of AntiOxP, pointing out that DCM could extract good antioxidant compounds from bio-oil. The pyrolytic lignin fraction (WI-DCMI) also led to a similar value of AntiOxP, but in this case with a lower soluble dosage in biodiesel, which highlights the good antioxidant properties of some lignin-derived macromolecules present in this fraction.

AntiOxP values were noticeably different from each other when comparing the performance of the bio-oil subfractions separated by preparative-SEC. Among the WS-DCMS subfractions, WS-DCMS-M (smallest molecular size) showed the best antioxidant properties, followed by WS-DCMS-D. However, WS-DCMS-T hardly showed an effect on the oxidation stability of biodiesel. Therefore, size fractionation was especially useful to isolate and concentrate good antioxidant compounds initially contained in WS-DCMS fraction: AntiOxP reached a mean value of 82 with WS-DCMS-M, while this parameter was 50 with the parent WS-DCMS fraction.

Among the WI-DCMS subfractions, WI-DCMS-D showed the highest AntiOxP parameter (higher than WI-DCMS-M), which was attributed to the higher content of aromatic hydroxy group (3.96 mmol Ar–OH/g in WI-DCMS-D vs 3.14 mmol Ar–OH/g in WI-DCMS-M). The subfractions with higher molecular masses (WI-DCMS-H with a *M*_w_ of 1803 and WI-DCMS-T with a *M*_w_ of 1542, Table 2) showed lower antioxidant potential. Both fractions presented good solubility in biodiesel (ASD values of 0.79 and 0.50, respectively) but low content in aromatic hydroxy groups (1.72 mmol Ar–OH/g in WI-DCMS-T, Table SI-4), which finally led to a low value of the AntiOxP (8 and 22, respectively).

This is not the case with the WI-DCMI fraction, which was poorly soluble in biodiesel ASD of 0.2 but presented a remarkable AntiOxP value (51), in the same order as those obtained with WS-DCMS-D or WI-DCMS-D (41). One clear difference between these heavy subfractions is the number of aromatic hydroxy groups, which was significantly higher in WI-DCMI (3.54 mmol Ar–OH/g), thus contributing to the poor solubility of this fraction in the hydrophobic biodiesel (ASD of 0.2), but also enhancing the antioxidant capacity of the solubilized fraction. Consequently, because of this high number of aromatic hydroxy groups and low solubility, WI-DCMI presented a relatively high AntiOxP value.

In summary, the antioxidant properties of bio-oil fractions are closely related to their chemical structure. For instance, fractions rich in monomeric phenolics, such as WS-DCMS-M, exhibited higher antioxidant activity due to the presence of hydroxyl groups capable of donating hydrogen atoms to neutralize free radicals. Conversely, larger molecular weight fractions containing more complex structures may have limited solubility and lower interaction with biodiesel, affecting their overall efficacy.

The evolution of AntiOxP has been compared against the content in aromatic hydroxy groups (Ar–OH) measured by ^31^P-NMR, showing a close relationship. Thus, when bio-oil (1.18 mmol Ar–OH/g, AntiOxP = 16.7) was solvent fractionated, WI-DCMS (2.92 mmol Ar–OH/g, AntiOxP = 52) and WS-DCMS (2.78 mmol Ar–OH/g, AntiOxP = 50) were far more active against biodiesel oxidation. A similar trend was observed upon molecular weight fractionation, in which Ar–OH groups were concentrated in WS-DCMS-M (3.96 mmol Ar–OH/g, AntiOxP = 82) and WI-DCMS-D (3.96 mmol Ar–OH/g, AntiOxP = 41).

Among phenolic compounds, the catechol group (benzene with two aromatic hydroxy groups) is known to have good antioxidant properties.^51^ In this work, the catechol group was identified to some extent in all samples, and broadly, higher values of AntiOxP correlate with higher concentrations of catechol units. However, the evolution of the AntiOxP parameter cannot be fully explained by only considering catechol units, especially in the case of the monomer-rich fractions. The WI-DCMS-M did not show the highest AntiOxP value despite having the highest content of catechol, which could be explained by synergic or even antagonist effects of other compounds in the fractions.

To further discuss the effect of the different functionalities on the AntiOxP of each fraction, Pearson statistical analyses were performed to assess the correlation between the AntiOxP and each one of the functionalities quantified in the samples by ^13^C-NMR (Table 3) and ^31^P-NMR (Table SI-4 in the Supporting Information). A Pearson coefficient (PC) of 1 means a perfect positive correlation between variables, while a value of −1 means a perfect negative correlation. The statistical analysis did not reveal a significant effect of the molecular size of the phenolics on their antioxidant power, which would be related just to the presence of certain functional groups. Analyzing more in-depth the hydroxy groups listed in Table SI-3, the AntiOxP parameter exhibited a positive effect (p-values lower than 0.05) with the aromatic (PC = 0.823), guaiacyl (PC = 0.804) and catechol (PC = 0.640) moieties. The high correlation with total aromatic-C simply indicates that those moieties with antioxidant activity are joined to an aromatic structure. Within phenolic functionalities, the presence of guayacyl and catechol ones would positively affect the antioxidant power of the fraction, while no effect of syringyl moieties was found in the analysis. There are multiple free radical scavenging mechanisms involving catechol or guaiacyl moieties, and the preferred mechanisms vary depending on the reaction phase.^52^ According to the literature, guayacyl and catechol functionalities prefer to trap free radicals by multiple Hydrogen Atom Transfer (HAT) mechanisms in a benzene phase (which could be similar to biodiesel in terms of intermolecular forces).^52^ These authors claimed that the strong intramolecular hydrogen bonds that can be formed in catechol moiety can negatively influence the antioxidant activity of its hydrogen atom/proton-donating group, which would justify the higher PC obtained in this work for the correlation of the guaiacyl group concentration and the AntiOxP. Thus, to produce more efficient antioxidants, further upgrading of bio-oil should seek these functionalities.

Finally, to have a first approach on how the bio-oil fractions compare with the synthetic phenolic compounds currently used to prevent biodiesel oxidation, Table 5 shows the comparative experimental data obtained for biodiesel doped with BHT at different dosages.

**Table 5:** Oxidation Stability Tests of Biodiesel Doped with BHT

**sample**	**ASD-wt %**	***t***_neat BD_**(min)**	***t***_doped BD_**(min)**	**AntiOxP**
BD (BHT)_1	0.87	20.5 ± 0.1	110.0	99.0
BD (BHT)_2	0.66	20.5 ± 0.1	93.4	107.6
BD (BHT)_3	0.44	20.5 ± 0.1	74.1	120.1

In general, the antioxidant performance of BHT was significantly better than the bio-oil additives prepared in this work, but in some cases the results were comparable, especially for the fraction WS-DCMS-M. For the same soluble dosage of 0.44 wt %, the oxidation test lasted for 59 min with the WS-DCMS-M subfraction, while it lasted up to 74 min with BHT. This makes a difference of only 25% in terms of oxidation stability time, which highlights the potential of this fraction to be exploited as an antioxidant additive for biofuels.

## 4 Conclusions

The research findings suggest that bio-oil fractions obtained from pine wood pyrolysis hold potential as a valuable source of antioxidants. Through the use of solvent extraction (initially with water and subsequently with dichloromethane) and size-exclusion chromatography (SEC), the bio-oil underwent fractionation into distinct fractions, each demonstrating varying chemical functionalities, molecular sizes and antioxidant capacities (assessed through the enhancement of biodiesel oxidative stability).

The experimental results revealed that solvent fractionation initially provided a size-based separation: the compounds of the water-insoluble dichloromethane-insoluble fraction (WI-DCMI) demonstrated higher molecular masses (mainly between 934 and1755 Da) compared to those of the crude bio-oil sample (186–271 Da), while the water-soluble dichloromethane-soluble compounds (WS-DCMS) displayed predominantly lower values (154–262 Da).

Monomeric phenols were effectively extracted from the bio-oil utilizing dichloromethane. Then, preparative-SEC of these extracted fractions facilitated their concentration in low molecular weight subfractions, with the best case achieving a concentration as high as 82% of phenolics (approximately 5% yield relative to crude bio-oil). The antioxidant potential of this subfraction significantly outperformed that of the crude bio-oil, being approximately five times more effective. Furthermore, oligomeric phenolic structures were scarcely found in the heaviest subfractions separated from WI-DCMS or WS-DCMS, but appeared to remain in the WI-DCMI fraction. Despite its reduced solubility in biodiesel, this fraction also demonstrated commendable antioxidant performance. The increase in aromatic hydroxy content (quantified by ^31^P-NMR), particularly guaiacol and catechol functionalities, was closely associated with the antioxidant effectiveness of these fractions, unlike the molecular weight, which did not yield a significant effect.

In summary, the study demonstrates that specific bio-oil fractions, particularly those rich in monomeric phenolics, can significantly enhance the oxidative stability of biodiesel. These findings provide a foundation for developing new and sustainable antioxidant additives from bio-oil, which can improve biodiesel’s life and performance, thereby supporting the industry’s move toward greener and more sustainable fuel solutions.

## Supporting Information Available

Detailed description of the bio-oil fractionation procedure (Figure SI-1). Detailed description of the characterization by gas chromatography coupled with mass spectrometry and flame ionization detection (GC/MS/FID) of bio-oil fractions. Peaks of maximum wavelength emission for various model compounds in UV-fluorescence (Table SI-3). Detailed description of the characterization by the Nuclear Magnetic Resonance (NMR) of bio-oil fractions. SEC chromatograms (RID) and DOSY spectra for bio-oil (BO), WI-DCMS, WS-DCMS and WI-DCMI (Figure SI-2). HSQC spectra for crude bio-oil and WS-DCMS in DMSO-d^6^ (Figure SI-3). DOSY-NMR spectra for WS-DCMS and WI-DCMS and their size-subfractions in the 0–10 ppm range (Figure SI-4). ^13^C-NMR spectra for bio-oil, WI-DCMS and WS-DCMS, and their size-subfractions (Figure SI-5). WS-DCMI characterization: DOSY spectra, ^13^CIG spectra, SEC chromatogram (with RID and with UV–vis) (Figure SI-6). Quantification of hydroxy groups by ^31^P-NMR in the different bio-oil fractions and subfractions (Table SI-4).(PDF)
Calculation of the RRF for each compound used for quantification of the FID signal in the GC/MS/FID characterization (XLSX)
List of the compounds identified and quantified by GC/MS/FID in the different fractions and subfractions of bio-oil (XLSX)
