# Supplementary material for: Bio-oil Fractionation According to Polarity and Molecular Size: Characterization and Application as Antioxidants
Source: Energy Fuels. 2024 Sep 25;38(19):18688–704. doi: 10.1021/acs.energyfuels.4c02641 (PMC11460040; doi:10.1021/acs.energyfuels.4c02641)
Supplement: Supplementary file 1 — ef4c02641_si_001.pdf [file ef4c02641_si_001.pdf]

SUPPORTING INFORMATION

Bio-oil fractionation according to polarity and molecular size: characterization and application as antioxidants

Isabel Fonts (0000-0001-7035-1955)[a], Cristina Lázaro[a], Alfonso Cornejo (0000-0001-8810-0062)[b], José Luis Sánchez (0000-0002-9705-2207)[a], Zainab Afailal (0000-0002-9091-672X)[a], Noemí Gil-Lalaguna (0000-0002-8704-9274)[a*], Jesús María Arauzo (0000-0002-5959-3168)[a]

[a] University of Zaragoza, Aragon Institute for Engineering Research (I3A), Thermochemical Processes Group (GPT), 50018, Zaragoza, Spain

[b] Public University of Navarra, Institute for Advanced Materials and Mathematics (INAMAT^2) - Department of Sciences, 31006, Pamplona, Spain

*Corresponding author: noemigil@unizar.es

S1. Bio-oil fractionation procedure

Solvent extraction. First, bio-oil was fractionated by solvent extraction with water and subsequently with dichloromethane (DCM) following an adapted method from a widely used bio-oil characterization procedure [1]. In the first step, approximately 25 g of bio-oil was mixed with deionized water in a 1:10 mass ratio to obtain a water-soluble (WS) fraction and an insoluble one (WI) separated by filtration. The WS fraction was subsequently extracted with 250 mL of DCM to obtain a water-soluble/DCM-insoluble (WS-DCMI) and a water-soluble/DCM-soluble (WS-DCMS) fraction. The WI fraction was also extracted with DCM (250 mL), yielding two more fractions, soluble and insoluble in DCM (WI-DCMS and WI-DCMI), which were separated by filtration. Both solvents, water and DCM, were removed by rotary distillation at 40 °C to determine bio-oil mass distribution among the different fractions (except for the WS-DCMS one, which is known to contain low molecular weight compounds that could be easily lost during evaporation, so its mass percentage was calculated by the difference between the whole liquid and the rest of the fractions, also including the water content of bio-oil). According to the literature [1], the WS-DCMI fraction contains the most polar compounds, such as anhydrosugars, low molecular weight acids and hydroxy acids. The WS-DCMS contains, among others, aldehydes, ketones and phenolic monomers. Once separated and dried, the WI-DCMI becomes a brown powder. In the literature, this fraction is usually referred to as high molecular weight pyrolytic lignin and its average molecular mass (1050 Da) is sensibly higher than that of the WS-DCMS fraction (Oasmaa et al., 2003b). GC-eluted compounds of the WI-DCMS fraction are poorly water-soluble lignin monomers (guaiacol derivatives) and lignin dimers (stilbenes) [1].

Size fractionation of DCM-soluble fractions by preparative size exclusion chromatography (SEC). DCM-soluble fractions were subjected to preparative-SEC using a Puriflash 5.125 (Interchim, France) equipped with an Omnifit column (25 mm diameter and 50.5 cm long). The stationary phase consisted of a Bio-Bead S-X3 resin (Bio-Rad Laboratories, USA) that was swollen in DCM overnight. The sample injection was performed in liquid form using a homemade loop

of 0.50 mL and a 6-way valve. The equipment has an automatic system for fraction collection (132 tubes, 20 mL each) and a UV detector (200-400 nm).

Three phenolic model compounds (phenol (94.1 g/mol), 2,2'-Methylenebis(6-tert-butyl-4-methylphenol) (340.5 g/mol) and 1,3,5-Trimethyl-2,4,6-tris(3,5-di-tert-butyl-4-hydroxybenzyl)benzene (775.2 g/mol)) were used for setting elution time intervals according to molecular size. The best resolution was achieved using a DCM solution with 10 wt.% of each phenolic compound (total concentration of 30 wt.%) at a 2.75 mL/min flow rate.

Once the method had been optimized, the WS-DCMS and WI-DCMS fractions were subjected to the preparative-SEC procedure to separate different molecular weight subfractions to be characterized and evaluated according to their antioxidant abilities. Both phases were previously dried and redissolved in DCM to adjust their concentration to 30 wt.% dissolutions.

Figure SI-1 summarizes the whole fractionation scheme, showing the different bio-oil fractions obtained after solvent extraction and preparative-SEC.

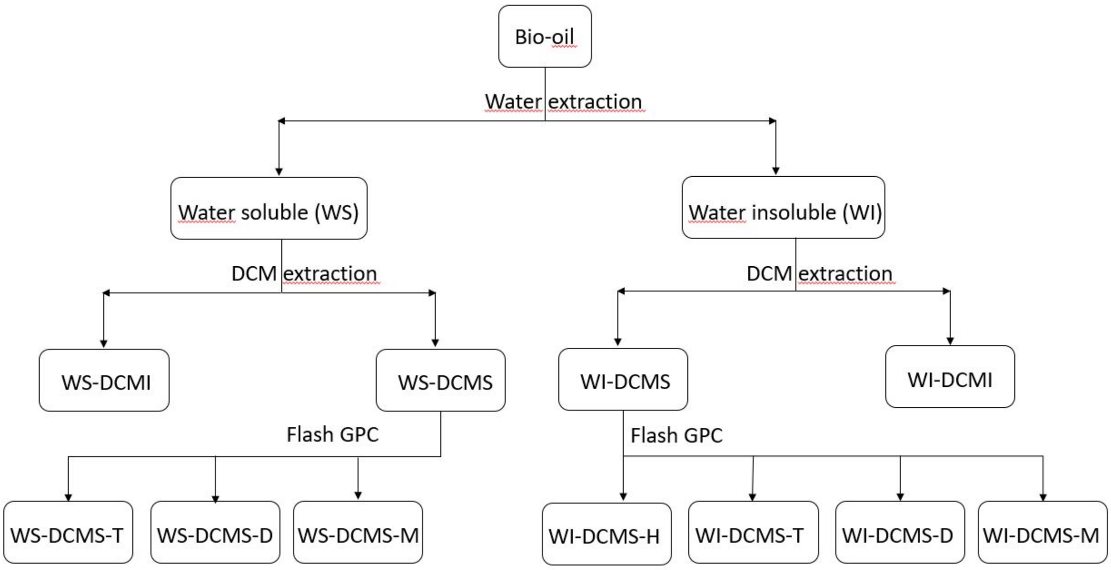

Figure SI-1. Bio-oil fractionation scheme.

S2. Chemical characterization of the bio-oil fractions

Gas chromatography coupled with mass spectrometry and flame ionization detection (GC/MS/FID). An Agilent GC/MS/FID (7890A/5975C) equipped with an Agilent DB-17MS column (30 m long, 0.250 mm diameter and 0.25 µm film thickness) was used to analyze the volatile compounds. For better identification and quantification, samples were previously derivatized by silylation with N,O-Bis(trimethylsilyl)trifluoroacetamide (CAS 25561-30-2; Sigma-Aldrich) according to the following procedure: 100 µL of each sample (dissolved in DCM) were introduced into a chromatography vial and dried in a stream of N_2 at 40 ^{0}C for 15 min. Then, 200 µL of the silylation reagent was added to the vial and kept at 80 °C for 30 min. Once cooled, the derivatized solution was dried, and the solid residue was dissolved in 100 µL of dichloromethane for GC/MS/FID analysis. 1 µL of each silylated sample was injected in the GC/MS/FID chromatograph using the splitless mode. The GC followed a temperature program starting at 50 °C (maintained for 2 min), after which the column was heated at 320 °C (5 °C/min) and held

for 10 min. Identification of compounds was performed with the MS signal using spectra in the NIST14 library, whereas the FID signal was used for the quantification using relative response factors (RRF) calculated according to the Effective Carbon Number for silylated compounds [2]. The RRF of toluene was assumed to be 1 and used as a reference for calculating the RRF of the rest of the compounds. The calculation procedure and the calculated RRF values of the compounds identified in the fractions and subfractions are shown in Table SI-1 (excel file). RRF were applied, assuming that all the samples' compounds were identified and all of them were volatile (assuming that the identified compounds were the only components in the fractions, which could be expected for the subfractions with lower molecular weight). Table SI-2 (excel file) shows the quantification results as percentage data.

Synchronous excitation UV-fluorescence spectroscopy (UV-fluorescence)

Table SI-3. Peaks of maximum wavelength emission for various model compounds in UV-fluorescence.

Compound	CAS	Molecular weight (Da)	$\lambda_{\text{max emisión}}$ (nm)
Phenol	Cas 108-95-2	94.11	278.28
Guaiacol	Cas 90-05-1	124.14	284.2
p-Cresol	Cas 106-44-5	108.14	285
Creosol	Cas 93-51-6	138.16	290
Eugenol	Cas 97 53 0	164.2	290.76
Dimer: 2,2′-Methylenebis(6-tert-butyl-4-methylphenol)	Cas 119-47-1	340.50	285.78
Tetramer: 1,3,5-Trimethyl-2,4,6-tris(3,5-di-tert-butyl-4-hydroxybenzyl)benzene	Cas 1709-70-2	775.20	283.28
Depolymerized argan			285.62, 329.38
Organosolv lignin			286.4, 328.3

Nuclear Magnetic Resonance (NMR). These analyses were made at 300 K on a Bruker Ascend III spectrometer equipped with a PH-BBI 5 mm probe, at 400 MHz and 101 MHz for ^{1}H and ^{13}C, respectively and were processed using Bruker Topspin 3.6.2 software. NMR samples were prepared in DMSO-d_6 and referenced using the residual signal at 2.50 ppm and 39.52 ppm for ^{1}H and ^{13}C measurements. ^{1}H experiments were run using the *zg30* pulse program at 16 scans. Quantitative ^{13}C-NMR samples were prepared dissolving *ca.* 50 mg in DMSO-d_6 (500 μL), and the spectra were acquired using an inverse gated decoupling pulse, *zgig30* program, that acquired 50000 scans with D1 = 2.0 s, using trimethylsilylpropilsulfonate sodium salt as an internal standard which was used to confirm that the integration was quantitative. Two-dimensional ^{1}H-^{13}C correlation was carried out using Heteronuclear Single Quantum Coherence spectroscopy (HSQC) and Heteronuclear Single Quantum Coherence-Total Correlation spectroscopy (HSQC-TOCSY) that run *hsqcetgpsi2* and *hsqcdietgpsisp.2* pulse programs in an echo-antiecho acquisition mode with D1 = 1.48 s and D1 = 2.0 s, respectively. DOSY was run using *stebpgp1s* pulse program in QF acquisition mode. Diffusion delay (d20) in *stebpgp1s* was optimized for each experiment using residual DMSO signal, keeping gradient pulse length (p30) constant at 1000 μs, resulting in 160-170 ms. Each pseudo-2D experiment consisted of a series of 16 spectra.

^{31}P-NMR measurements were made using a proton-decoupled experiment running the *zgpg30* pulse program. The samples were derivatized before ^{31}P measurements following the protocol

described by Pu et al. [3], using 2-chloro-4,4,5,5-tetramethyl-1,3,2-dioxaphospholane (TMDP) as a derivatizing agent in $CHCl_3$/ pyridine medium and N-hydroxy-5-norbornene-2,3-dicarboximide as an internal standard according to the method described by Ben and Ragauskas [4].

DOSY-NMR experiments were done in DMSO-d_6 at 300 K and a fixed low concentration (5.0 mg of sample in 500 μL of DMSO-d_6). The accuracy of the gradient was checked by the determination of the diffusion coefficient of residual DMSO in DMSO-d_6. DOSY analyses were processed using TOPSPIN 3.6.2 software from Bruker. Once F2 was phased, automatic baseline correction was run using a 5th-grade polynomial function. Using the T1/T2 relaxation module installed permitted FID (Free Induction Decay) processing for the first spectrum (2 % gradient) to be extracted and to perform manual integration. The integration regions were exported to the relaxation module, where decay values were fitted by area using the *vargrad* preinstalled function and 5.35 G/mm as gradient calibration constant. Graphical processing was run using 'Dynamic Center v. 2.6.1' software from Bruker. Unless otherwise stated, the Stejskal-Tanner equation was fitted using the intensity obtained after the peak picking of the first spectrum corresponding to a 2% gradient. In previous work, the calibration of the diffusion coefficient of polystyrene (PS) and polyethylene glycol and monomeric phenols (PEGP) standards and their corresponding molecular weights was described [5]. This calibration was successfully used to determine the mass of the aromatic and aliphatic fractions arising from lignin depolymerization.

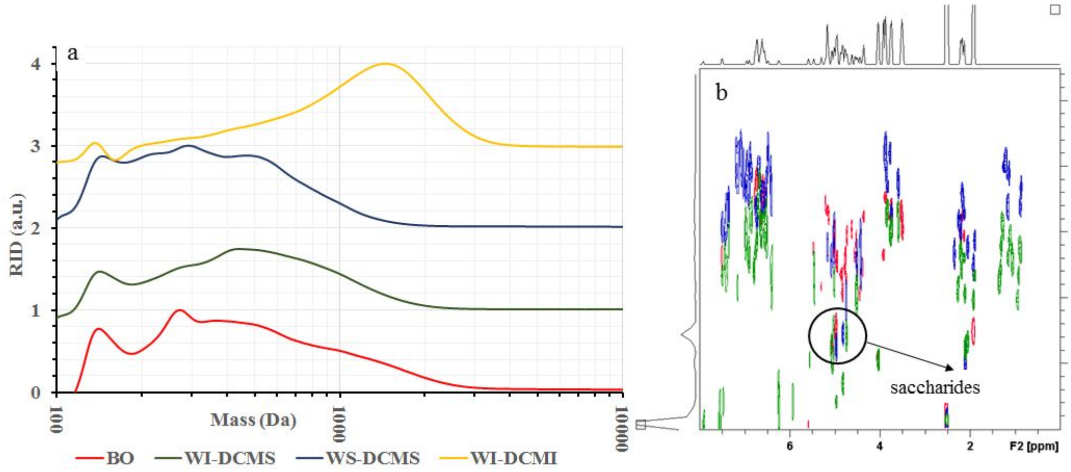

Figure SI-2. a) SEC chromatograms obtained with RID detector for bio-oil (BO), WI-DCMS, WS-DCMS and WI-DCMI; b) DOSY spectra for bio-oil (red), WI-DCMS (green) and WS-DCMS (blue).

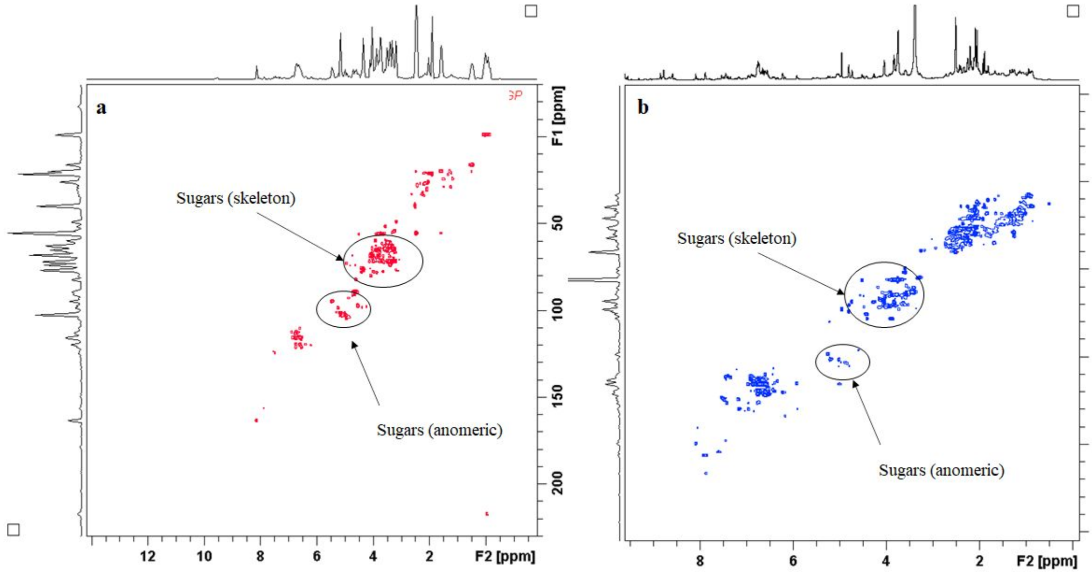

Figure SI-3. HSQC spectra for (a) crude bio-oil and (b) WS-DCMS in DMSO-d6

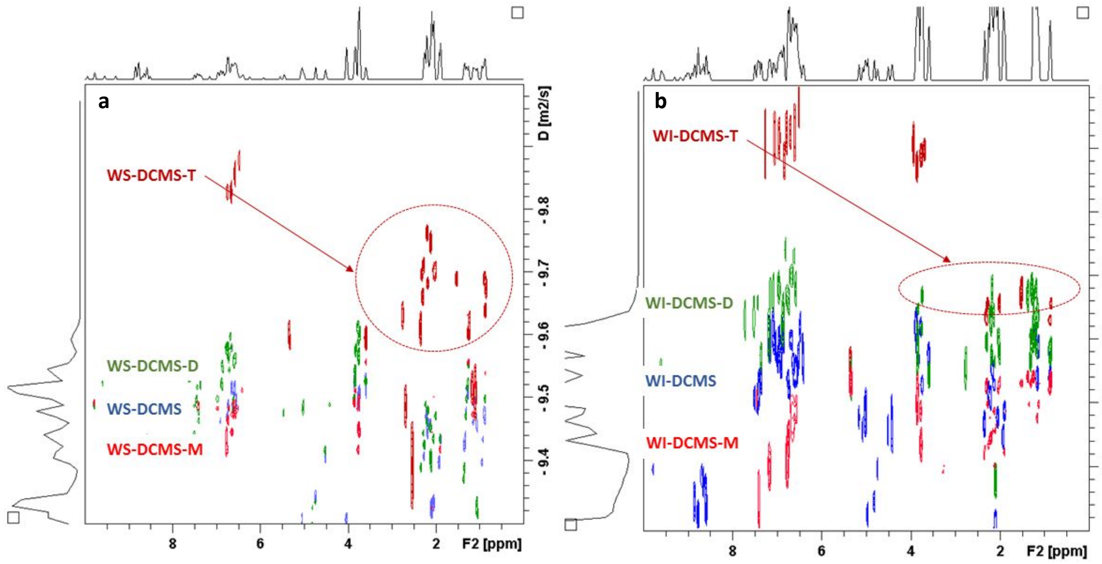

Figure SI-4. DOSY-NMR spectra for (a) WS-DCMS and (b) WI-DCMS and their size-subfractions in the 0-10 ppm range.

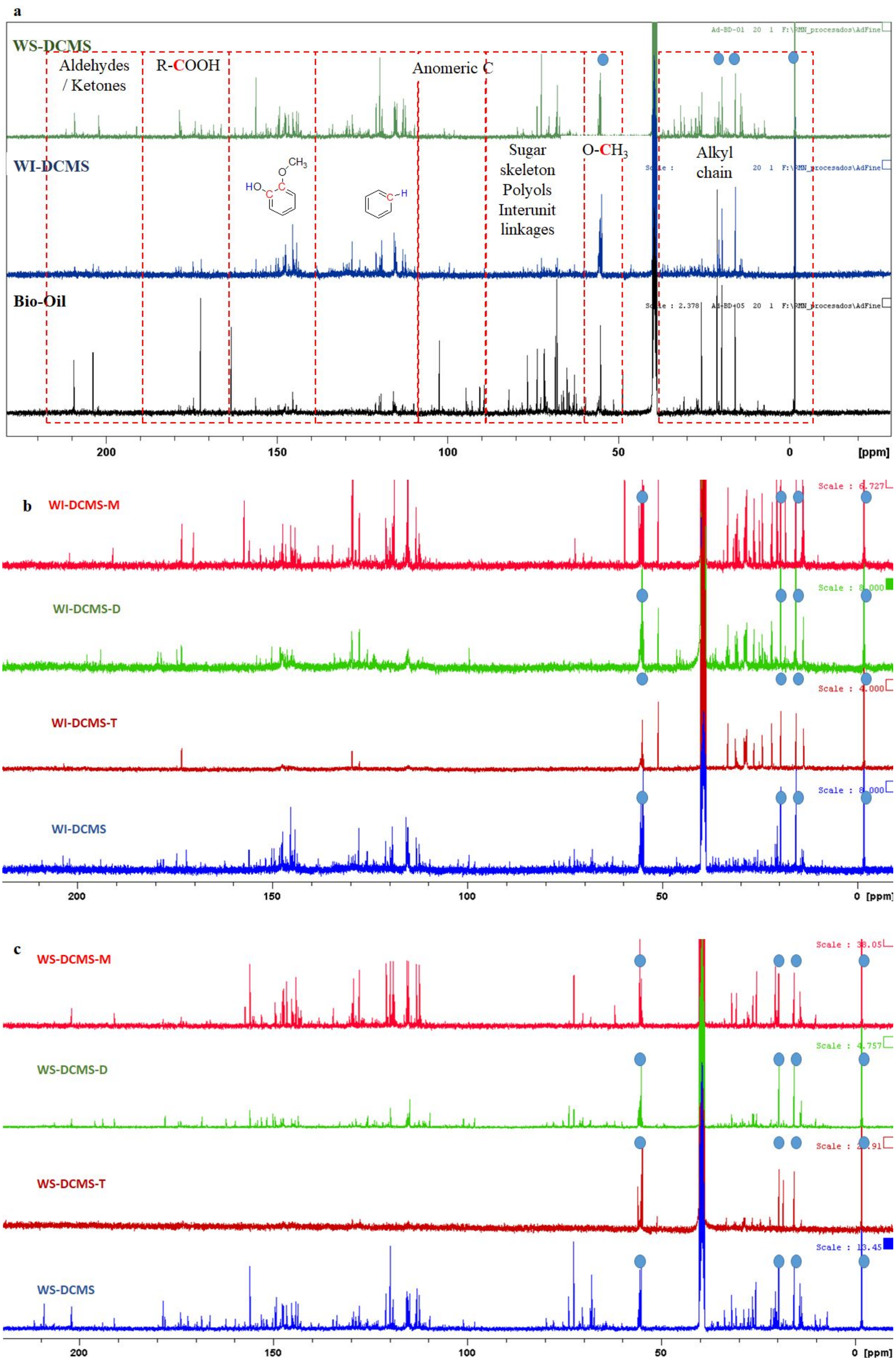

Figure SI-5. ^{13}C-NMR spectra for (a) bio-oil, WI-DCMS and WS-DCMS; b) WI-DCMS size-subfractions and b) WS-DCMS size-subfractions. (*Blue circles denote internal standard signals*).

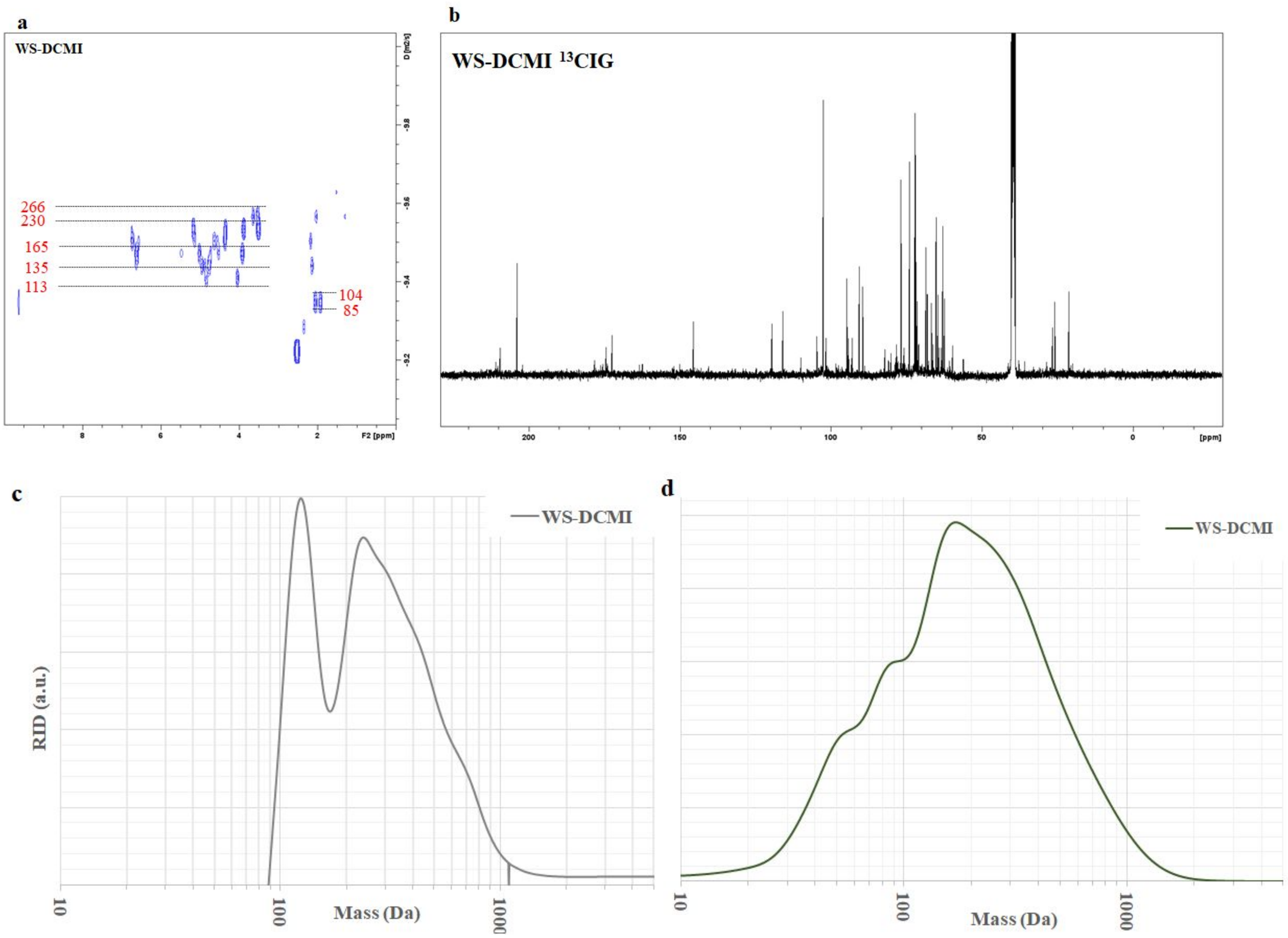

Figure SI-6. a) DOSY spectra for WS-DCMI. b) ^{13}CIG spectra for WS-DCMI. c) SEC chromatogram (RID) for WS-DCMI. d) SEC chromatogram (UV-VIS, λ=254 nm) for WS-DCMI.

Table SI-4. Quantification of hydroxy groups by ^{31}P-NMR.

	Hydroxyl group (mmol/g)						
**Sample**	**Aromatic**	**Aliphatic**	**Syringyl**	**Guaiacyl**	**Cathecol**	**Acidic**	**Total**
Bio-oil	1.18	2.77	0.08	0.25	0.30	0.57	3.95
WI-DCMI (Rep 1)	3.37	1.59	0.34	0.76	0.93	0.26	4.96
WI-DCMI (Rep 2)	3.54	0.88	0.40	0.77	0.91	0.28	4.41
WS-DCMS (Rep 1)	2.78	1.62	0.18	0.96	0.76	0.54	4.40
WS-DCMS (Rep 2)	2.72	1.54	0.1	0.72	1.17	2.1	4.26
WI-DCMS	2.92	1.57	0.27	1.09	0.91	0.59	4.50
WS-DCMS-T	0.91	0.57	0.07	0.28	0.24	0.07	1.48
WS-DCMS-D	2.43	1.37	0.12	0.8	0.79	0.37	3.81
WS-DCMS-M	3.96	0.08	0.17	1.66	1.21	0.08	4.04
WI-DCMS-T	1.72	0.32	0.18	0.53	0.42	0.03	2.04
WI-DCMS-D	3.96	0.08	0.17	1.66	1.21	0.08	4.04
WI-DCMS-M	3.14	0.08	0.18	0.69	1.51	0.1	3.23

References:

[1] Oasmaa, A., Kuoppala, E., & Solantausta, Y. (2003a). Fast pyrolysis of forestry residue. 2. Physicochemical composition of product liquid. *Energy and Fuels*, *17*(2), 433–443. https://doi.org/10.1021/ef020206g

[2] De Saint Laumer, J. Y., Leocata, S., Tissot, E., Baroux, L., Kampf, D. M., Merle, P., Boschung, A., Seyfried, M., & Chaintreau, A. (2015). Prediction of response factors for gas chromatography with flame ionization detection: Algorithm improvement, extension to silylated compounds, and application to the quantification of metabolites. *Journal of Separation Science*, *38*(18), 3209–3217. https://doi.org/10.1002/JSSC.201500106

[3] Pu, Y., Cao, S., & Ragauskas, A. J. (2011). Application of quantitative 31P NMR in biomass lignin and biofuel precursors characterization. Energy & Environmental Science, 4(9), 3154–3166. https://doi.org/10.1039/C1EE01201K

[4] Ben, H., & Ragauskas, A. J. (2011). NMR characterization of pyrolysis oils from kraft lignin. *Energy and Fuels*, *25*(5), 2322–2332. https://doi.org/10.1021/ef2001162

[5] Cornejo, A., García-Yoldi, Í., Alegria-Dallo, I., Galilea-Gonzalo, R., Hablich, K., Sánchez, D., Otazu, E., Funcia, I., Gil, M. J., & Martínez-Merino, V. (2020). Systematic Diffusion-Ordered Spectroscopy for the Selective Determination of Molecular Weight in Real Lignins and Fractions Arising from Base-Catalyzed Depolymerization Reaction Mixtures. *ACS Sustainable Chemistry and Engineering*, *8*(23), 8638–8647. https://doi.org/10.1021/acssuschemeng.0c01375
